# Donor‐influenced Structure–Activity Correlations in Stoichiometric and Catalytic Reactions of Lithium Monoamido‐Monohydrido‐Dialkylaluminates

**DOI:** 10.1002/chem.201801541

**Published:** 2018-06-13

**Authors:** Lara E. Lemmerz, Ross McLellan, Neil R. Judge, Alan R. Kennedy, Samantha A. Orr, Marina Uzelac, Eva Hevia, Stuart D. Robertson, Jun Okuda, Robert E. Mulvey

**Affiliations:** ^1^ WestCHEM Department of Pure and Applied Chemistry University of Strathclyde Glasgow G1 1XL UK; ^2^ Institute of Inorganic Chemistry, RWTH Aachen University Landoltweg 1 52056 Aachen Germany

**Keywords:** aluminate, homogeneous catalysis, hydroboration, lithium, metallation

## Abstract

A series of heteroleptic monoamido‐monohydrido‐dialkylaluminate complexes of general formula [*i*Bu_2_AlTMPHLi⋅donor] were synthesized and characterised in solution and in the solid state. Applying these complexes in catalytic hydroboration reactions with representative aldehydes and ketones reveals that all are competent, however a definite donor substituent effect is discernible. The bifunctional nature of the complexes is also probed by assessing their performance in metallation of a triazole and phenylacetylene and addition across pyrazine. These results lead to an example of phenylacetylene hydroboration, which likely proceeds via deprotonation, rather than insertion as observed with the aldehydes and ketones. Collectively, the results emphasise that reactivity is strongly influenced by both the mixed‐metal constitution and mixed‐ligand constitution of the new aluminates.

## Introduction

Well‐defined main‐group metal complexes are currently the subject of burgeoning synthetic interest, in both stoichiometric and catalytic transformations.^1^ This attention stems from the realisation that, as chemists, we need to develop new sustainable solutions without recourse to scarce and toxic noble metals, whilst at the same time attempting to emulate their renowned reactivity. Furthermore, the plethora of earth abundant main group metals requires that we need to develop a more fundamental understanding of the potential and the limits of mono‐ and bimetallic main group metal systems. In this regard aluminium, the most abundant metal in the earth's crust, fits the requirement. Reports of aluminium complexes in important stoichiometric and catalytic processes are becoming increasingly common in the literature. For example aluminium reagents are emerging as important reagents in (catalyst free) cross‐coupling protocols^2^ and in metallation.^3^ In the latter case, we recently reported that heteroleptic *i*Bu_2_AlTMP in tandem with LiTMP can metallate a range of *sp* ^2^‐ and *sp* ^3^‐C−H bonds, albeit the presence of the bulky TMP anion bound to lithium is crucial in the C−H bond activation.3f–3k In fact it is only in rare cases with relatively acidic hydrogen atoms that aluminium reagents in isolation have demonstrated utility in deprotonative metallation of organic C−H substrates.1a In the catalytic arena vis‐à‐vis hydroboration, the use of aluminium complexes is gaining momentum.^4^ Importantly, Roesky et al. utilized a β‐diketiminato stabilised aluminium hydride complex in hydroboration of alkynes and carbonyl groups.4a Recently, the groups of Cowley and Thomas demonstrated that the commerically available DIBAL(H) or Et_3_Al⋅DABCO are capable of catalysing the hydroboration of alkynes.4c At this point, most aluminium based catalysts have been neutral complexes, though recent reports have implicated borates as important species in hydroboration.^5^

Since our groups interest lie in the synergistically beneficial interplay of two distinct metal centres in a bimetallic “ate” complex, this prompted the question whether alkali‐metal aluminates would demonstrably impart exciting reactivity to hydroboration chemistry. Therefore, in a recent communication we reported a range of Lewis donor solvated lithium aluminates bearing two HMDS (1,1,1,3,3,3‐hexamethyldisilazide) and two hydride functionalities and established that lithium diamidodihydridoaluminates were able to function efficiently in hydroboration catalysis and metallation applications.^6^ Very recently, following their aforementioned alkyne hydroboration advances, Cowley and Thomas have been successful in hydroboration of alkenes using the commercial ates, LiAlH_4_ and sodium bis(2‐methoxyethoxy)aluminum hydride.^7^ In our lithium diamidodihydridoaluminates cases the nature of the supporting Lewis donor (level of solvation, N‐donor versus O‐donor) played an influential role in catalytic performance, where easily displaced monodentate donors performed better than the polydentate donors, presumably because the chelating effect proved to be deleterious, blocking the active metal sites. Moreover, we also observed similar phenomena in a related catalytic dehydrocoupling process using the metal hydride surrogate, dihydropyridine precatalyst, 1‐Li‐2‐*t*Bu‐1,2‐dihydropyridine.^8^ The Okuda group also recently reported a series of alkali metal hydridotriphenylborates,^9^ that showed the nature of the Lewis donor (including flexibility of coordination and consequential Lewis acidity of the metal) impacted hydroboration performance in an unpredictable manner. Thus, in main group (bi)metallic catalysis, even small changes in the nature of ancillary ligand(s) impart large differences in the ensuing reactivity. Given this, here we have examined for the first time structure–activity relationships of donor solvated heteroleptic dialkyl‐monoamido‐monohydrido complexes using TMP (2,2,6,6‐tetramethylpiperidide) as the amide of choice. TMP is a superior base to its HMDS counterpart and is arguably the most important utility amide having taken over from diisopropylamide,^10^ on account of its widespread employment not only in LiTMP but in a series of bimetallic formulations such as Knochel's salt‐supported magnesium and zinc reagents^11^ and the organometallic ate type reagents introduced by Kondo/Uchiyama/Wheatley, Mongin, and ourselves.^3^, ^12^ Thus, we report here the synthesis of a series of these new aluminates, investigate their solid state and solution structures and compare their reactivity in hydroboration of aldehydes and ketones, and assess their ambi‐utility in metallation and addition reactions.

## Results and Discussion

### Synthesis and characterisation

Cocomplexation between the commercially available aluminium hydride DIBAL(H) and LiTMP in *n*‐hexane at room temperature resulted in the immediate precipitation of a white powder **1** that so far has resisted recrystallisation thus ruling out X‐ray crystallographic authentication. However, NMR spectroscopic studies have revealed its general constitution. ^1^H and ^13^C spectra reveal the presence of two isobutyl groups and one TMP group and the hydride ligand. The ^7^Li spectrum displays a sharp resonance at *δ*=0.4 ppm albeit the ^27^Al spectrum does not contain any identifiable resonance, a common problem with quadrupolar aluminium centres in broadly unsymmetrical environments. The presence of an aluminium hydride was confirmed by performing a ^1^H{^27^Al} experiment which revealed a sharpening of the broad resonance at 3.18 ppm, and suggested formation of the expected cocomplex *i*Bu_2_AlTMP(H)Li **1** in a 75 % isolated yield. Structural insight of the solution phase constitution of **1** was gained by performing a DOSY experiment in C_6_D_6_ solution,^13^ revealing the likely aggregation state of the solvent‐free **1** as dimeric with the formula [*i*Bu_2_AlTMP(H)Li]_2_. Similar solvent‐free structures are known in the literature and contain the common [Li_2_H_2_] central square core depicted in **1**.^14^

**Scheme 1:**
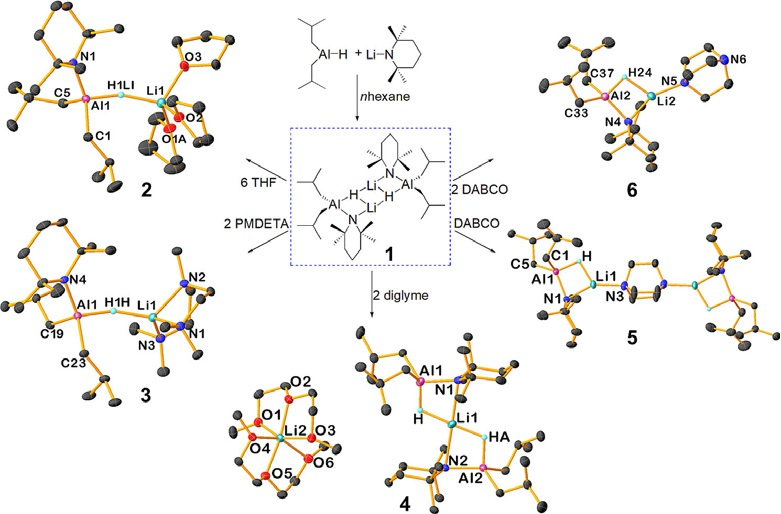
Synthesis of complexes **1**–**6**, including postulated structure of powder **1** and molecular structures of crystalline **2**–**6**. All hydrogen atoms other than hydrido types bonded to aluminium are omitted for clarity. Thermal ellipsoids are drawn at 30 % probability.

Next, a series of donor‐solvated derivatives of **1** were obtained in high yield by addition of the appropriate ligand (THF, PMDETA, diglyme or DABCO) into toluene solutions of **1**, followed by introduction of *n*‐hexane and crystallisation at −30 °C (Scheme 1). In each case, single‐crystals suitable for X‐ray diffraction studies were obtained and revealed a remarkable variance in the molecular arrangements. THF adduct **2** and PMDETA adduct **3** in effect exhibit the same structure, wherein a distorted tetrahedral *i*Bu_2_AlTMP(H) fragment is bonded, via the μ‐hydride, to a Li⋅donor fragment (donor=three THF in **2** and one PMDETA in **3**). Both the Al−H and Li−H distances are similar in each molecule [Al−H 1.61(3) Å in **2**, 1.66(4) Å in **3**; Li−H 1.83(3) Å in **2**, 1.76(4) Å in **3**], with those to the group 13 metal systematically shorter by an average of 0.16 Å. Changing the Lewis donor from PMDETA to diglyme gives **4**, which adopts a markedly different solid‐state structure. The main differences between these tridentate chelating ligands is a change from N to O donors, and importantly the latter only contains one terminal methyl substituent, reducing steric congestion when it ligates a metal atom. Charge‐separated ion pair structure **4** is best described as a lithium lithium‐dialuminate, since both cationic and anionic moieties contain lithium. The cationic moiety is a distorted octahedral lithium cation supported by two diglyme ligands. The anionic moiety is comprised of two peripheral *i*Bu_2_AlTMP(H) units in distorted tetrahedral environments, that bond to a central lithium via μ‐TMP and μ‐hydride ligands. Furthermore the lithium ion displays a near square planar geometry (τ_4_=0.04),^15^ albeit it is also likely to be further stabilised by electrostatic interactions from TMP methyl substituents [Li−C_Me_ range: 2.946(7)–3.249(7) Å]. This arrangement can be described as an inverse Weiss motif. The Weiss motif is a surprisingly common structure found in dialkali‐metal “ate” complexes where the central, non‐alkali‐metal exists in a tetrahedral arrangement, with respect to four bridging ligands.^16^ Here in contrast we have a central square planar alkali‐metal that bridges through ligands to two peripheral aluminium atoms. A search for this structural motif in the Cambridge Structural Database (CSD) resulted in zero entries, confirming the structural rarity of **4**. Reaction of **1** with half an equivalent of DABCO affords **5** in which two symmetry equivalent *i*Bu_2_AlTMP(H)Li subunits are connected by the bicyclic, binitrogen Lewis donor. As with **4**, here the lithium ion coordination sphere is completed by μ‐TMP and μ‐hydride ligands. Employing a full equivalent of DABCO affords the stoichiometric variant **6**, which differs from **5** by a terminal DABCO ligand, instead of the bridging mode observed in **5**. In **2**–**6**, despite the diversity of donor ligands, the Al−H and Li−H distances do not display any systematic differences across the series (Al−H range 1.61(3)–1.69(3) Å; Li−H range 1.76(4)–1.88(3) Å). The structures of **2**–**6** and the related lithium diamidodihydridoaluminates we reported previously,^6^ can be regarded as well‐defined modifications of LiAlH_4_ containing a range of either alkyl or amido groups in place of either two or three hydrogen atoms. Furthermore, these more substituted LiAlH_4_ modifications benefit from the synthetic advantages of enhanced solubility (soluble in hydrocarbon solvents), and easier to accurately weigh low loadings in catalytic applications.

**Figure 1:**
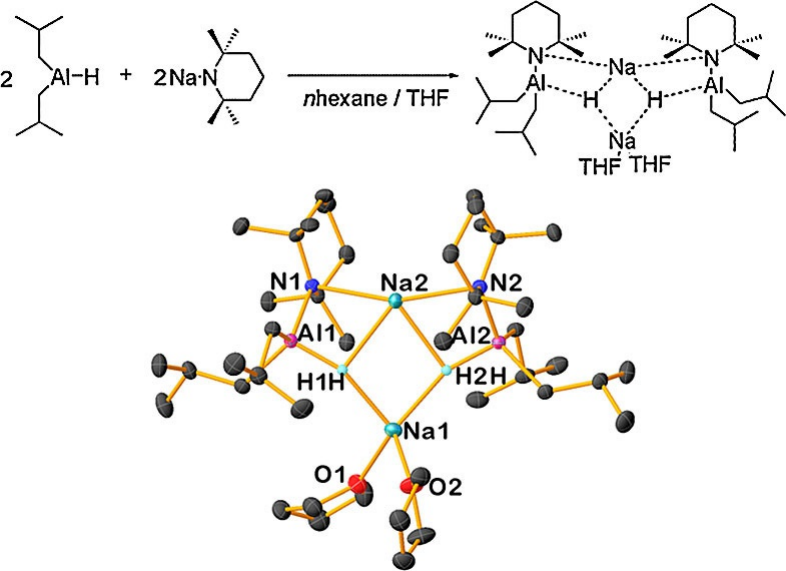
(top) Synthesis of **7**. (bottom) Molecular structure of **7**. All hydrogen atoms other than the hydrido types bonded to aluminium are omitted for clarity. Thermal ellipsoids are drawn at 30 % probability.

In the knowledge that alkali metal effects can profoundly influence structural morphology, reactivity and physical properties,^12^, ^17^ we next attempted to synthesise a sodium variant by reaction of NaTMP with DIBAL(H) in *n*‐hexane. After addition of a small amount of THF to the reaction mixture we were able to isolate a few crystals of (*i*Bu_2_Alμ‐TMPμ_3_‐HNa)_2_⋅2THF, **7**, that were found to be suitable for diffraction studies (Figure 1). Crystallographic characterisation of **7** revealed a structure markedly different from the lithium congeners, highlighting the impact of replacing lithium with its larger congener. Figure 1 shows an arrangement wherein a (NaHNaH) near‐planar kite shaped ring lies between two *i*Bu_2_AlTMP in an asymmetric fashion. One sodium is supported by two μ‐TMP and two μ_3_‐hydride ligands (Na2−H1H 2.40(2) Å and Na2−H2H 2.38(2) Å). The second sodium is also bonded to the hydride ligands [Na1−H1H 2.22(2) Å and Na1−H2H 2.19(2) Å] and two solvating THF molecules. Unfortunately, we were unable to reproducibly prepare **7** in acceptable yields and as such its characterisation remained restricted to this structural analysis.

**Figure 2:**
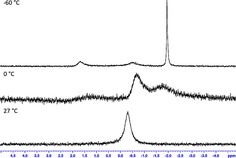
Variable temperature ^7^Li NMR study of **4** in [D_8_]toluene solution.

Complexes **2**–**6** were also characterised in solution by ^1^H, ^13^C, ^7^Li and ^27^Al NMR spectroscopy and revealed the expected resonances, albeit as with **1**, the ^27^Al spectra were of little diagnostic value. ^1^H and ^13^C spectra confirmed the presence of 2 *i*Bu, 1 TMP, and an aluminium bound hydride ligand, as well as the appropriate donor resonance(s). Interestingly the ^1^H NMR spectrums of **5** and **6** are near identical, except for the chemical shift resonances of the DABCO CH_2_ protons. Moreover, in **6** the terminally bound DABCO ligand only contains one broad singlet instead of two triplets (vide infra). The ^7^Li NMR spectrum of **4** displayed one noticeably broad resonance at *δ*=−0.32 ppm, instead of the expected two resonances in accordance with the charged‐separated molecules in the crystal. Thus, a variable temperature ^7^Li NMR experiment was performed to investigate whether an exchange process is occurring (Figure 2). Measuring a ^7^Li NMR spectrum of **4** in a [D_8_]toluene solution at 0 °C results in a significant broadening compared with the room temperature collection. Further cooling at temperatures down to −60 °C results in the appearance of two broad and one sharp resonance at *δ*=1.68, −0.36 and −1.96 ppm, indicating the likelihood of an exchange process occurring at room temperature. The identity of the three resonances can be tentatively assigned. Those at *δ*=1.68 and −1.96 can be assigned to a structure that resembles that in the solid state, with the latter sharper resonance corresponding to the approximately octahedral Li⋅(diglyme)_2_ cation. The resonance at −0.36 ppm may be assigned to a contact ion pair resembling PMDETA analogue **3**, which exhibits a sharp resonance at *δ*=0.38 ppm. We probed this exchange process further via a DOSY NMR study of **4** at room temperature in C_6_D_6_. The estimated molecular weights in this instance are 509 and 615 g mol^−1]^ (note the Lewis donor resonances diffuse separately from the remaining resonances), which are intermediate between those of either the expected mass in the crystal (846 g mol^−1^) or a structure resembling **3** (424 g mol^−1^). In contrast, DOSY NMR studies of **3** indicate its greater robustness as no such exchange process occurs on the NMR timescale, where an estimated molecular weight (463 g mol^−1^) is in excellent agreement with the theoretical value (446 g mol^−1^).

In summary, complexes **1**–**6** represent a series of new monoamido‐monohydrido lithium aluminates, well defined in the solid and/or solution states. Key distinctions are **1** is donor free; **3** contains a solvating PMDETA ligand in the solid state and retains this arrangement in solution (via DOSY studies); **4** adopts an entirely novel solid‐state arrangement vide supra, and exhibits exchange of the diglyme ligands on the NMR timescale.

### Hydroboration of aldehydes and ketones

At the inception of these studies we sought to discover the answer to two key questions: (i) can these monoamido monohydrido lithium aluminates act as efficient catalysts in hydroboration applications; and (ii) does the structure, determined by the donor ligand play a role in any such catalytic performance? In this regard we initially selected **1**, **3**, and **4**, since **1** is donor‐free, while **3** and **4** contain broadly similar donor ligands, except for the donor atom identity (N or O), and the slightly reduced steric profile of diglyme which gives rise to a fluxional system in solution. Hydroboration catalysis using HBpin was selected to trial **1**, **3** and **4**, since it is an area currently attracting increasing interest in the main‐group arena, and the wider chemical audience.^18^ Importantly, simple aluminium reagents (AlEt_3_⋅DABCO, DIBAL(H), LiAlH_4_)4c, 7 have recently been discovered as excellent catalysts in this regard, although occasionally the presence of a second metal has been overlooked when considering the elementary steps of the catalytic profile. Given our groups’ longstanding association with bimetallic “ate” complexes, we pondered whether these systems might also shed further light on the reaction pathways and any significant effect of the second metal. Results of catalytic hydroboration of aldehydes and ketones are presented in Table 1.

**Table 1:** Hydroboration of aldehydes and ketones using **1**–**6**.^[a]^

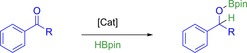			
Substrate	Catalyst	Time [h]	Yield [%]
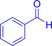	**1** ^[d]^ **3** ^[d]^ **4** ^[d]^	0.25 0.25 0.25	99 99 99
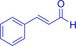	**1** **2** **3** **4** **5** **6**	0.5 0.5 3 0.5 0.5 0.5	99 99 99 99 98 98
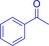	**1** **1** ^[b]^ **2** **3** **3** ^[c]^ **3** ^[b]^ **4** **5** **6**	2.5 0.5 0.5 65 22 0.5 2 0.75 0.75	97 95 93 99 99 94 90 96 96
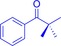	**3**	2.5	98
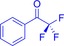	**3**	0.25	99
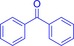	**1** **3** **4**	0.5 3 0.5	99 99 99
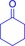	**1** **3** **4**	2 3 1.5	93 99 99
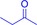	**1** **3** **4**	1.5 3.5 3.5	99 98 97

[a] Reaction conditions (unless stated otherwise): in C_6_D_6_ at room temperature. NMR conversions determined with respect to 10 mol % of hexamethylcyclotrisiloxane as internal standard. Catalyst loadings are all 5 mol % based upon Al/Li present in the solid‐state structure. [b] In [D_8_]THF. [c] At 70 °C. [d] 1 mol % catalyst loading

Reaction of **1** (2.5 mol % based upon a dimeric formula), **3** (5 mol %) or **4** (2.5 mol %) with benzaldehyde and pinacol borane in C_6_D_6_ solution resulted in fast and quantitative hydroboration in 15 minutes at room temperature in every case. Lowering the precatalyst loadings to 0.5 mol % of **1** or **4** and 1 mol % of **3** resulted in no loss of catalytic performance. Importantly these initial results demonstrate that **1**, **3** and **4** are all suitable candidates for aldehyde hydroboration. Expanding the substrate scope to include dual‐functional cinnamaldehyde demonstrates catalyst selectivity with smooth and quantitative hydroboration occurring only at the carbonyl functionality. **1** and **4** achieve this transformation inside 30 min, whereas with **3** quantitative hydroboration occurs after 3 h. This demonstrates that subtle structural differences within these systems play a role in catalyst performance. This performance is broadly in line with previous aluminium‐based hydroboration catalysts. Roesky et al. achieved quantitiative hydroboration of cinnamaldehyde with his nacnacAlH_2_ complex in 6 h with 1 % loading, whereas we previously demonstrated 76 % conversion in 2 h with (HMDS)_2_AlH(μ‐H)Li(THF)_3_. Completing our studies into aldehyde hydroboration we screened complexes **2**, **5** and **6** with cinnamaldehyde as a representative example. **2** contains a four coordinate lithium atom albeit bonded to three monodentate THF ligands, which are more likely to desolvate during the reaction, compared to tridentate PMDETA in **3**, thus substrate access to lithium may occur more readily. **5** and **6** both have coordinately unsaturated three coordinate lithium atoms, and as such are readily accessible. Complexes **2**, **5** and **6** all efficiently catalyse hydroboration of cinnamaldehyde in 30 min.

**Scheme 2:**
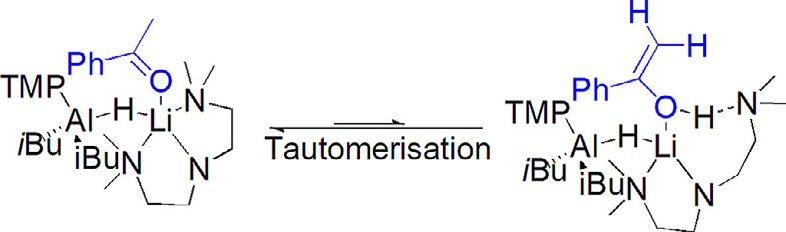
Keto–enol tautomerisation of acetophenone as a possible contribution towards the slow catalytic performance observed with **3**.

Encouraged by these findings we extended our substrates to include ketones, rationalising the increased intrinsic steric bulk compared to that of aldehydes would magnify any ligand effects in these systems. Hydroboration of acetophenone with **1** (2.5 mol %) reaches 97 % conversion after 2.5 h at room temperature in C_6_D_6_. Using **3** as precatalyst, the reaction requires 65 h for quantitative hydroboration at room temperature, or 22 h at 70 °C. Using **4** this reaction takes 2 h for 90 % conversion, similar to the value using **1**. Exploring the comparatively poor performance of **3** more thoroughly, 2,2,2‐trimethylacetophenone and 2,2,2‐trifluoroacetophenone were selected as substrates. The former shows enhanced steric features with respect to acetophenone, whereas the latter, approximately isosteric, is considerably more electron withdrawing. Hydroboration of 2,2,2‐trimethylacetophenone with **3** (5 mol %) occurs in 2.5 h at room temperature, implying that ligand steric features are not the only factor here in determining hydroboration efficiency. 2,2,2‐trifluoroacetophenone is quantitatively hydroborated in 15 min at room temperature. This fast reactivity may be expected since the presence of CF_3_ would significantly deplete the charge present at the ketone carbon atom making it more electrophilic and thus facilitate faster nucleophilic hydride insertion. One possible contribution towards the slow reactivity of **3** with acetophenone is that the presence of α‐hydrogen atoms may promote keto‐enol tautomerism under the reaction conditions. In this scenario, one may envisage a coordination of the substrate to the lithium ion, resulting in a sterically congested 5‐coordinate lithium ion, followed by tautomerisation and stabilisation of the enol form by H‐bonding to one nitrogen atom of the PMDETA ligand (Scheme 2). However, it is expected that the keto‐form would predominate in solution, and we detect no spectroscopic evidence of its enol tautomer. Note no such tautomerisation and H‐bond stabilisation is possible with the CF_3_ analogue. A second suggestion for the slow catalytic transformation of acetophenone with **3** is that a methyl hydrogen atom may be deprotonated by the TMP group. However, since this reaction eventually results in quantitative hydroboration, any deprotonation must be under equilibrium.

Hydroboration catalysis of **1**, **3** and **4** with benzophenone again reveals that **3** performs less efficiently (3 h for quantitative formation) than either **1** or **4** (30 min). Using cyclohexanone as substrate reveals that **3** is again slowest (3 h); whereas **4** performs marginally better than **1** (1.5 h versus 2 h) for this reaction. Finally, butan‐2‐one, an aliphatic ketone, is fastest using **1** as a catalyst (1.5 h), while both **3** and **4** are complete in 3.5 h at room temperature. Butan‐2‐one contains two sets of α‐hydrogens which may explain the slower reactivity with respect to **1**. Hydroboration of acetophenone was also performed using **2**, **5** and **6**. All three complexes demonstrate essentially the same reactivity (**2**, 30 min, 93 % conversion, **5** and **6**, 45 min, quantitative). This can be rationalised by considering the structural distinctions of the complexes. In each case the DABCO ligand is monodentate, rendering the lithium atom 3‐coordinate, instead of the fully saturated 4‐coordinate in **2**–**4**, albeit the high lability of the solvating THF in **2** is likely to lower the coordination number as required during reaction. Therefore, one may anticipate that initial coordination of substrate to lithium, and thus subsequent insertion into the Al−H bond would occur more readily in these cases. Importantly this scenario validates the use and future exploration of bimetallic systems.

The general trend observed between **1**, **3**, and **4** in these reactions reveal that **1**, the donor free complex is the best catalyst and marginally outperforms **4. 3** in all cases is the least efficient catalyst in these processes. This reactivity can be related to the solid and solution phase structures. The structure of **3** in solution, via DOSY NMR studies, resembles that in the crystal structure. Thus, one expects the PMDETA group with its three donor atoms to remain tightly bound to lithium during the catalytic process. On the other hand, **4** can be considered a halfway‐house between **1** and **3**. Spectroscopic studies indicate that part of the time in solution the complex resembles that of the solid‐state structure, which is donor free, akin to **1**, (both diglyme ligands are involved in bonding to the separated lithium cation). The remainder of the time the complex likely resembles **3**, and thus in general, delivers reactivity intermediate between that of **1** and **3**. A lower lithium coordination number also plays a role in catalytic performance as indicated by reactivity of **2**, **5** and **6** which hydroborate acetophenone faster than the other donor solvates studied. Once again, we turned to DOSY NMR studies to illuminate the solution constitution of these complexes to gain insight into the catalytic process. The estimated molecular weight of **2** is 486 g mol^−1^, in good agreement with the theoretical value (506 g mol^−1^), suggesting that **2** largely remains intact in solution. However, careful inspection of the spectrum reveals that the THF resonances diffuse slightly faster than the remaining resonances of **2** (MW 444 g mol^−1^), detailing that in solution some level of THF desolvation is in operation, lowering the metal coordination number. With **5** and **6** the solution constitution is ambiguous. Bridging DABCO complex **5** has an estimated molecular weight of 620 g mol^−1^, lower than the expected value of 691 g mol^−1^. Similarly terminally bound DABCO complex **6** has an estimated molecular weight of 514 g mol^−1^, higher than the expected value of 402 g mol^−1^. These intermediate values indicate that the solution constitutions may be in equilibria between the two crystallographically observed extremes, and moreover, account for the similarity in ^1^H NMR spectra of **5** and **6** (vide supra). In each case the DABCO resonances diffuse with the same coefficient as the remaining complex resonances, ruling out complete ligand desolvation, affording **1**, on the NMR experiment timescale. Extending this argument one step further, we performed two representative reactions using bulk THF as reaction solvent. We selected **1** and **3** for these reactions and discovered that in each case quantitative hydroboration occurs within 30 min. These fast reactions can be tentatively attributed to, in the case of **1**, breaking up of the dimeric aggregate into a monomeric solvent separated species, and in the case of **3** displacing the PMDETA ligand resulting in a similar more labile solution species, an arrangement that favours faster catalytic transformation. Corroborating this, the ^1^H NMR spectrum of **3** in [D_8_]THF solution reveals the presence of free PMDETA.

**Scheme 3:**
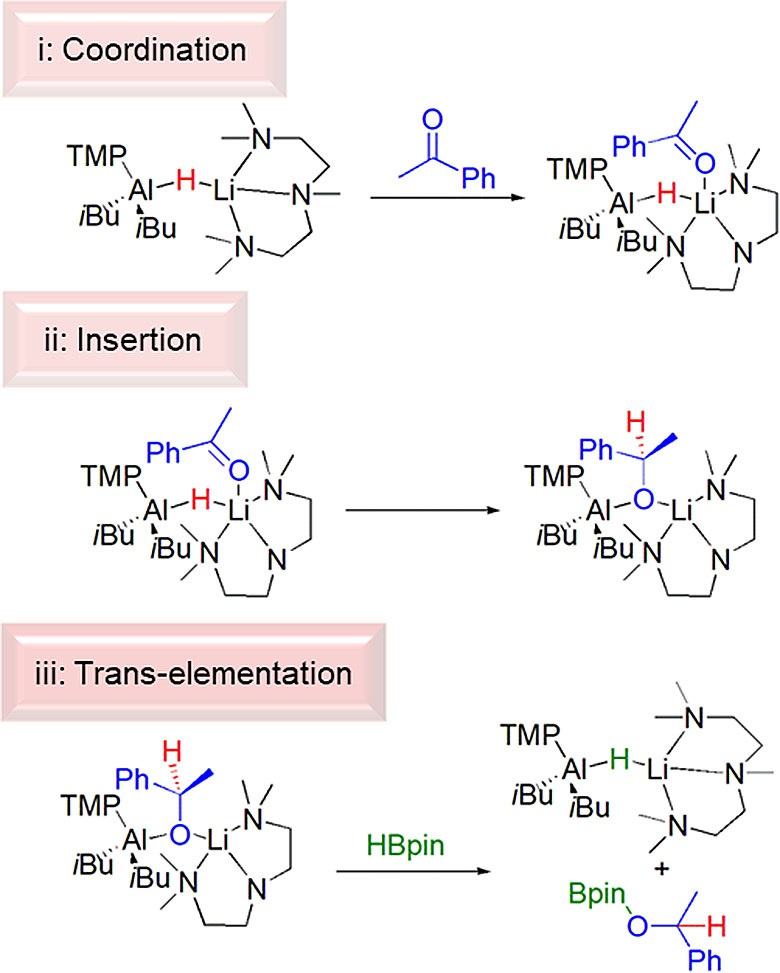
Representation of possible elementary steps using **3**: coordination of substrate; insertion into Al−H bond and σ‐bond metathesis with pinacol borane.

Following from the hydroboration results we sought to discover some mechanistic evidence by performing a series of stoichiometric reactions between complexes **1** or **3** with various substrate molecules. Unfortunately, we were unable to crystallographically characterise such a species, despite repeated attempts. However, the different hydroboration results, that is, the effect of the donor ligand leads us to surmise that hydroboration of aldehydes and ketones in C_6_D_6_ follows three basic elementary steps: (i) coordination of carbonyl substrate to lithium. Here, tentative evidence for the coordination step originates from the slow performance of **3** with acetophenone, (see Scheme 2); (ii) insertion of carbonyl into aluminium hydride bond, already primed for addition; (iii) transelementation with HBpin to afford hydroborated product and regenerate an active aluminium hydride catalyst. Scheme 3 details the proposed elementary steps in the reaction.

### Metallation

**Scheme 4:**
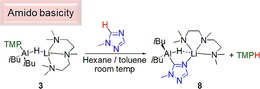
TMP basicity of **3** with 1‐methyl‐1,2,4‐triazole, affording **8**.

Complexes **1**–**6** all contain a basic TMP and a nucleophilic hydride ligand and all are competent, in varying degrees, in catalytic hydroboration transformations. Well‐defined PMDETA complex **3** was tested, as a representative complex in metallation reactions with substrates containing an acidic hydrogen atom. Metallation of 1,2,4‐triazoles in general can be problematic and often requires low reaction temperatures, to prevent unwelcome fragmentation reactions of metallated (commonly lithiated) intermediate species.^19^ Reaction of **3** with 1‐methyl‐1,2,4‐triazole at room temperature for 1 h in a *n*‐hexane/toluene mixture, followed by cooling at −30 °C resulted in formation of colourless crystals suitable for X‐ray diffraction studies (Scheme 4).

**Figure 3:**
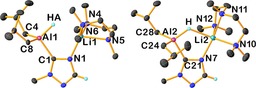
Molecular structures of the two crystallographically independent molecules in the asymmetric unit of **8**. All hydrogen atoms other than those bonded to aluminium, and the triazolyl C−H are omitted for clarity. Thermal ellipsoids are drawn at 30 % probability.

In agreement with the spectroscopic data (see Supporting Information) the X‐ray diffraction data revealed that the substrate has been metallated at the 5‐position, via amido basicity, giving **8** in a reasonable isolated yield (42 %). Heterotrileptic **8** (Figure 3) crystallises with two independent molecules in the asymmetric unit. In each case an *i*Bu_2_AlH unit bonds to the 5‐position of the triazole ring. Furthermore, the Al−H distances are the same within experimental error in each independent molecule 1.57(2) and 1.58(2) Å. A lithium⋅PMDETA moiety is held in place by coordination from the triazolyl nitrogen atom of the aluminate moiety placed adjacent to the metallated carbon atom. Further, in one independent molecule (RHS Figure 3) the hydride ligand bridges to Li [Li−H 2.23(2) Å], while in the second (LHS Figure 3) the Li−H distance is significantly longer [Li−H 2.40(3) Å]. Further inspection of the structure reveals a key factor for this drastic difference. In each case the PMDETA ligand coordinates to the lithium in a different manner. In one molecule (RHS) the methyl group attached to the central nitrogen atom lies approximately parallel to the Li−H interaction, whereas in the other (LHS) this same methyl group points in the opposite direction, a fact we attribute to packing effects in the crystalline lattice.

It is important to note that the aluminium reagent *i*Bu_2_AlTMP on its own can also metallate 1‐methyl‐1,2,4‐triazole. This reaction affords (*i*Bu_2_AlC_3_H_4_N_3_)_2_, **9** in 30 % isolated yield, and is a rare example of a neutral bis‐alkylamidoaluminium compound acting as an amido base towards an aromatic C−H bond, albeit with hydrogen atoms in relatively acidic environments.^20^ Though X‐ray crystallographic data for **9** were collected, these were of insufficient quality to justify publication (see Supporting Information).

**Scheme 5:**
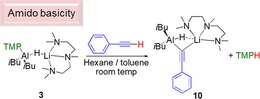
TMP basicity of **3** with phenylacetylene, affording **10**.

Phenylacetylene was selected as a candidate metallation substrate because of its acidic CCH hydrogen and because of precedence from Roesky's research that implicated deprotonation as a key step in terminal alkyne hydroboration.4b In that report the basicity derives from a hydride of a β‐diketiminato stabilised AlH_2_ unit. Using **3** (as a representative example, and the PMDETA ligand to try and induce crystallisation of the formed product) we sought to discover whether deprotonation or addition would occur, and whether any deprotonation would derive from the more basic TMP unit or weaker hydride, and whether these complexes could then be implicated in subsequent catalytic hydroboration reactions. Reaction between **3** and phenylacetylene in a J. Young's NMR tube was carried out at room temperature. ^1^H NMR monitoring revealed the appearance of resonances corresponding to TMPH. Furthermore, the resonance corresponding to the acidic hydrogen of phenylacetylene disappeared, confirming that **3** reacts, once again, as an amido base (Scheme 5). The reaction was repeated in a toluene/*n*‐hexane mixture at room temperature. After stirring the solution for 2 h at room temperature **10** was isolated in 57 % yield as an off‐white solid. Spectroscopic characterisation of isolated **10** corresponds to a mono(alkynyl) monohydrido lithium aluminate, *i*Bu_2_AlHCCPhLi⋅PMDETA. Its structure closely resembles that of **3** via formal replacement of a TMP anion with a phenylalkynyl anion as was the case with the triazolyl anion in **8**. Note similar structures are known in the literature, and Uhl reported dialkylaluminium alkynides adopting the bridging motif shown in **10**, moreover with less bulky alkyl groups, a crystallographically authenticated pi interaction between aluminium and the triple bond was identified.

By extension to the present system, such an interaction between the triple bond and the carbophilic aluminium centre may prime the complex for insertion.^21^ Importantly, **10** is similar to an in silico modelled active catalytic intermediate by Roesky et al., (a neutral β‐diketiminato Al‐alkynyl hydride) from which the B−H bond of pinacol borane adds across the triple bond, followed by addition of a second equivalent of phenyl acetylene to regenerate the active component. Since Roesky's neutral intermediate complex was found to be an excellent catalyst in alkyne hydroboration, the isolation of the bimetallic complex **7**, by simple deprotonation, afforded the opportunity to learn about the role of anionic aluminates in this context (note that in this report we have two *i*Bu ligands instead of a bulky β‐diketiminate).

**Scheme 6:**
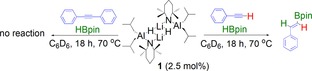
Attempted hydroboration reactions of diphenylacetylene (LHS) and phenylacetylene (RHS) using **1** as a precatalyst.

In a J. Young's NMR tube **1** (2.5 mol %) was dissolved in C_6_D_6_ to which phenylacetylene was added, followed by HBPin and the reaction monitored by ^1^H NMR spectroscopy. After heating for 18 h at 70 °C clean formation of the anti‐Markovnikov vinylboronate ester has occurred in 76 % yield, as referenced against an internal standard (Scheme 6). The experiment was repeated, with **3** instead of **1** as catalyst. In this case hydroboration only occurs very slowly, affording only around 5 % yield after heating at 70 °C for 18 hours.

**Scheme 7:**
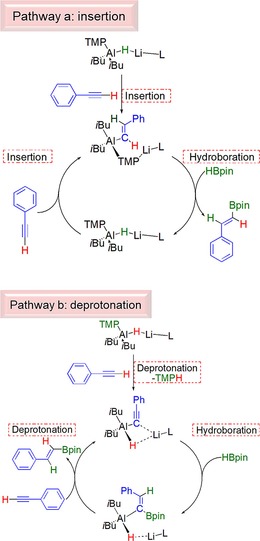
Alternative potential reaction pathways for lithium aluminate catalysed hydroboration of phenylacetylene.

This desirable outcome therefore suggests that well‐defined “ate” complexes can play an important role in hydroboration of a variety of unsaturated molecules. Scheme 7 outlines various potential reaction pathways in this process. Pathway a, in Scheme 7 describes an insertion pathway, that is a hydroalumination followed by a σ‐bond metathesis, analogous to that discussed (vide supra) for aldehyde and ketone hydroboration. Moreover, a recent report from Cowley and Thomas discuss a similar mechanism for the DIBAL(H) catalysed hydroboration of alkynes. Since deprotonation was observed in stoichiometric reactions between phenylacetylene and either **1** or **3**, we rationalise that this is a key step in any hydroboration catalysis of phenylacetylene, using the complexes herein. Interestingly, attempts to corroborate this observation proved unsuccessful. Reactions between **10**, or the PMDETA‐free variant, generated in situ, with HBpin both afforded ^1^H and ^11^B NMR spectra that were broadly uninterpretable, and indicative of decomposition of the reaction mixtures in stoichiometric regimes. A second proposal (Pathway b) proceeds in broad agreement to that of Roesky using a neutral NacNacAlH_2_ catalyst. Here, lithium aluminate **1**, performs an initial deprotonation, then hydroboration occurs, followed by a deprotonation of a second phenylacetylene molecule, inducing catalytic turnover.

Next, we decided to attempt the hydroboration of an internal alkyne. Reasoning that since di‐phenylacetylene did not contain any hydrogen atom “primed” for deprotonation, that any hydroboration activity would, by necessity, occur via an insertion of the alkyne into the aluminium hydride bond, and further shed light on the likely reaction pathway. Thus, diphenylacetylene was added to a J. Young's NMR tube in C_6_D_6_, followed by **1** (2.5 mol %) and HBpin. After heating the solution at 70 °C for 18 h the ^1^H NMR spectrum remained unchanged revealing that the addition of an Al−H across diphenylacetylene is not favoured, and gives credence to the reaction mechanism depicted pathway b in Scheme 7, although based on the results of stoichiometric reactions of **10** with HBpin, we cannot completely discount pathway a.

## Addition reactions

**Scheme 8:**
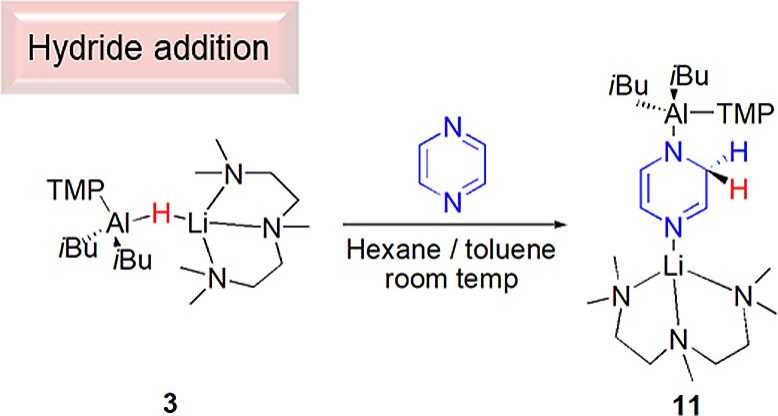
Reaction between **3** and pyrazine giving Al−H addition product **11**.

Despite the fact that addition of the Al−H bond did not freely occur across diphenylacetylene in the attempted hydroboration catalysis, we wanted to test whether addition reactions were a viable utility of these lithium aluminate complexes, thus we elected to react **3** with pyrazine. Both metallation and addition across pyrazine has previously been observed depending on the specific reagent(s) employed,^22^ prompting a consideration of whether the basicity of **3**, arising from the TMP ligand, or the nucleophilicity of the Al−H functionality would win out. A toluene/*n*‐hexane solution of **3** was stirred with one equivalent of pyrazine at room temperature for 2 h resulting in formation of a pale brown oil. ^1^H NMR spectra of the oil revealed clean formation of a single product, displaying four resonances between *δ*=7.3–4.0 ppm, in a 1:1:1:2 ratio consistent with hydride addition at the α‐carbon atom (Scheme 8). Importantly this result indicates that as well as being competent hydroboration catalysts, these complexes demonstrate great versatility as either metallating agents for acidic hydrogen atoms, or as H^−^ sources in addition reactions across pyrazine. Furthermore, it details the fine balance in these systems on the ensuing reactivity, which is highly substrate dependent.

## Conclusions

This contribution has demonstrated the synthesis, spectroscopic and structural characterisation of a new family of donor‐solvated heteroleptic dialkyl‐monoamido‐monohydrido complexes. The presence and nature of the Lewis donor imparts interesting structural characteristics that influence the catalytic activity in hydroboration reactions of aldehydes and ketones with pinacolborane. Polydentate donors (PMDETA) that remain bound to the lithium atom in solution slow down hydroboration. On the other hand, the related tridentate ligand diglyme, displays exchange on the NMR timescale, and performs similarly to the donor free species. We suggest this is, at least in part due to the coordination saturation of the lithium ion. Using the Lewis donor ligand DABCO leads to a reduction of the solvation at lithium and leads to faster hydroboration in a representative hydroboration of a ketone. In these catalytic transformations, insertion of the polar carbonyl group into the Al−H bond is mooted as a key step. Heteroleptic dialkyl‐monoamido‐monohydrido complexes are also revealed to be capable of hydroborating phenylacetylene, however this activity is suggested to proceed via deprotonation of the substrate. The bifunctional activity of the complexes is also demonstrated stoichiometrically in metallation of substrates containing an acidic hydrogen atom, and in an addition reaction with pyrazine.

## Experimental Section

Full experimental characterisation and synthetic procedures are described in the supporting information.

## Conflict of interest

The authors declare no conflict of interest.

## Supporting information

As a service to our authors and readers, this journal provides supporting information supplied by the authors. Such materials are peer reviewed and may be re‐organized for online delivery, but are not copy‐edited or typeset. Technical support issues arising from supporting information (other than missing files) should be addressed to the authors.

Supplementary
