# Supplementary material for: Donor‐influenced Structure–Activity Correlations in Stoichiometric and Catalytic Reactions of Lithium Monoamido‐Monohydrido‐Dialkylaluminates
Source: Chemistry. 2018 Jun 13;24(39):9940–8. doi: 10.1002/chem.201801541 (PMC6055685; doi:10.1002/chem.201801541)
Supplement: Supplementary file 1 — Supplementary [file CHEM-24-9940-s001.pdf]

CHEMISTRY
A European Journal

Supporting Information

Donor-influenced Structure–Activity Correlations in Stoichiometric and Catalytic Reactions of Lithium Monoamido-Monohydrido-Dialkylaluminates

Lara E. Lemmerz,[b] Ross McLellan,[a] Neil R. Judge,[a] Alan R. Kennedy,[a] Samantha A. Orr,[a] Marina Uzelac,[a] Eva Hevia,[a] Stuart D. Robertson,[a] Jun Okuda,*[b] and Robert E. Mulvey*[a]

chem_201801541_sm_miscellaneous_information.pdf

Donor-influenced structure-activity correlations in stoichiometric and catalytic reactions of lithium monoamido-monohydrido-dialkylaluminates

Lara E. Lemmerz,[b] Ross McLellan,[a] Neil R. Judge,[a] Alan R. Kennedy,[a] Samantha A. Orr,[a] Marina Uzelac,[a] Eva Hevia,[a] Stuart D. Robertson,[a] Jun Okuda,*[b] and Robert E. Mulvey*[a]

[a]WestCHEM, Department of Pure and Applied Chemistry, University of Strathclyde, Glasgow, G1 1XL, UK

[b] Institute of Inorganic Chemistry, RWTH Aachen University, Landoltweg1, 52056 Aachen (Germany)

General experimental considerations

All reactions and manipulations were conducted under a protective argon atmosphere using either standard Schlenk techniques or an MBraun glove box fitted with a gas purification and recirculation unit. NMR experiments were conducted in J. Youngs tubes oven dried and flushed with Argon prior to use. Solvents were dried by heating to reflux over sodium benzophenone ketyl and then distilled under nitrogen prior to use. All other reagents were purchased commercially from Sigma-Aldrich and used as received.

NMR Spectroscopy NMR spectra were recorded on a Bruker AV3 or AV 400 MHz spectrometer operating at 400.13 MHz for ^{1}H, 128.38 MHz for ^{11}B, 155.47 MHz for ^{7}Li, 100.62 MHz for ^{13}C and 104.2 MHz for ^{27}Al. All ^{13}C spectra were proton decoupled. ^{1}H and ^{13}C NMR spectra were referenced against the appropriate solvent signal. DOSY measurements were recorded on an AV 400 MHz spectrometer operating at 400.13 MHz, using the pulse program ledbpgp2s. In common with most organometallic molecules, estimated MW values have been calculated using the calibration curves for dissipated spheres and ellipsoids (DSE). TMS or tetraphenylnaphthalene have been used as references in the DOSY measurements.

X-ray Crystallography Crystallographic data were collected on Oxford Diffraction instruments with Mo Kα radiation (λ = 0.71073 Å) or Cu Kα.(λ = 1.54184) Structures were solved using SHELXS-97[1] or OLEX2,[2] while refinement was carried out on F2 against all independent reflections by the full matrix least-squares method using the SHELXL-97 program or by the GaussNewton algorithm using OLEX2. All non-hydrogen atoms were refined using anisotropic thermal parameters. Selected crystallographic details and refinement details are provided in table S1. CCDC 1833012-1833018 contains the supplementary crystallographic data for this structure. These data can be obtained free of charge from the Cambridge Crystallographic Data Centre via www.ccdc.cam.ac.uk/data_request/cif.

Catalytic hydroboration procedure

All reactions are carried out in 0.5 mL benzene-d_6 at room temperature (except where indicated otherwise) using 0.5 mmol of substrate and 0.55 mmol of HBpin. 10 mol% of hexamethylcyclotrisiloxane as an internal standard. Conversion was determined by ^{1}H NMR spectroscopy. In a typical experiment a J. Young's NMR tube was charged with substrate, NMR solvent and standard and the ^{1}H NMR spectrum recorded. HBpin and catalyst were added and the conversion was monitored over time by ^{1}H NMR spectroscopy.

Preparation of [LiHAlTMP(*i*Bu)$_2$]$_2$ (1): To a solution of *n*BuLi (3.13 mL, 5.0 mmol, 1.7M in *n*-hexane) in *n*-hexane (20 mL), 2,2,6,6-Tetramethylpiperidine (0.85 mL, 5.0 mmol, 1 equiv) was added dropwise, and the solution was stirred for 15 min. 5.0 mL of DIBAL (5.0 mmol, 1.0M in *n*-hexane) was added dropwise and the reaction mixture was stirred for 2 h; a colourless precipitate appeared after this time. The reaction suspension was reduced to a volume of 10 mL and the colourless solid was filtered off. [LiHAlTMP(*i*Bu)$_2$]$_2$ was isolated in 75% yield (1.09 g, 3.8 mmol). Note that **1** is only sparingly soluble in C_6D_6

^{1}H NMR (400.13 Hz, C_6D_6, 298 K) δ 3.18 (br. s, 1H, Li-H-Al), 2.21 (sept, 2H, AlCH$_2$C*H*(CH$_3$)$_2$), 1.50 (br. s, 2H, *p*-C*H*$_2$(TMP)), 1.32 (m, 24H, AlCH$_2$CH(C*H*$_3$)$_2$ and 4 x C*H*$_3$(TMP)), 1.15 (br. s, 4H, *m*-C*H*$_2$(TMP)), 0.55 (dd, 2H, $^3J_{HH}$ = 5.6 Hz, $^1J_{HH}$ = 13.5 Hz, AlC*H*$_2$CH(CH$_3$)$_2$), 0.38 (dd, 2H, $^3J_{HH}$ = 8.2 Hz, $^1J_{HH}$ = 13.5 Hz, AlC*H*$_2$CH(CH$_3$)$_2$) ppm.

^{13}C NMR (100.61 Hz, C_6D_6, 298 K) δ 44.4 (*m*-C*H*$_2$(TMP)), 32.9 (just observable in the HSQC, *C*H$_3$(TMP)), 29.5 (AlCH$_2$*C*H(CH$_3$)$_2$), 28.6 (observed in the ^{1}H-^{13}C HSQC spectrum, Al*C*H$_2$CH(CH$_3$)$_2$), 28.1 (AlCH$_2$CH(*C*H$_3$)$_2$), 28.0 (AlCH$_2$*C*H(CH$_3$)$_2$), 18.3 (*p*-*C*H$_2$(TMP)) ppm.

^{7}Li NMR (155.47 Hz, C_6D_6, 298 K) δ 0.04 (s, *Li*-H-Al) ppm.

Elemental analysis: *Calculated:* C: 70.55 %, H: 12.89 %, N: 4.84 %. *Found:* C: 70.09 %, H: 12.61 %, N: 4.99 %.

Figure S1: ^{1}H NMR spectrum of **1** in C_6D_6.

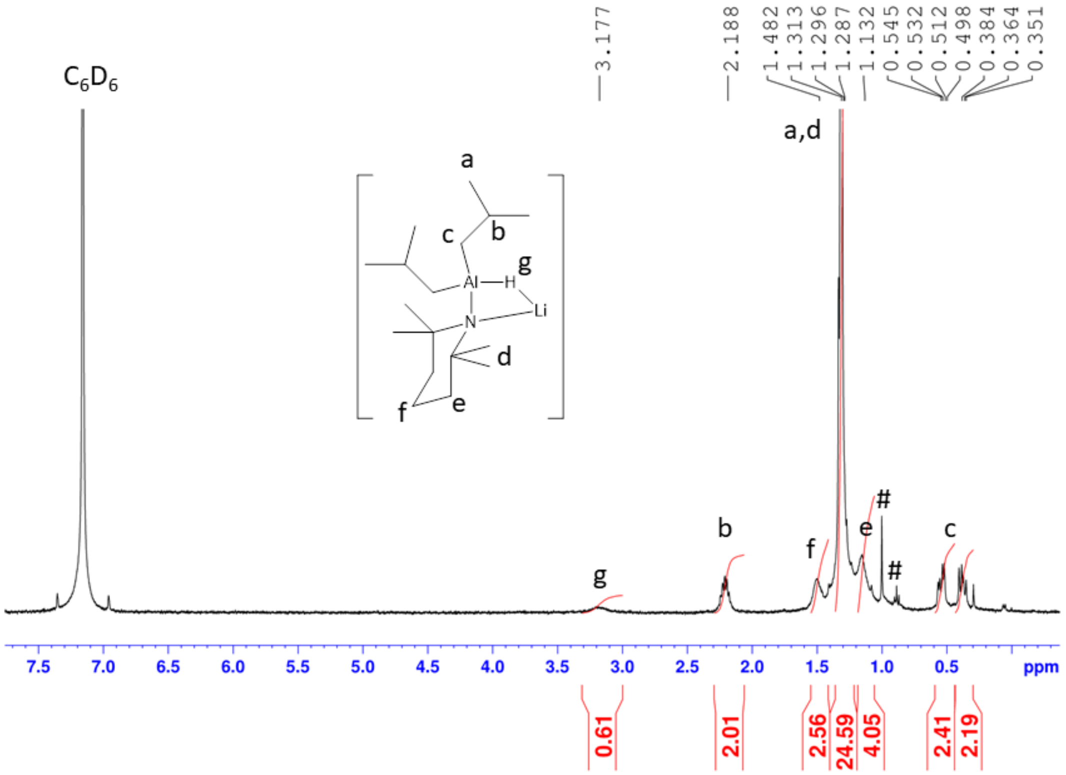

Figure S2: ^{13}C NMR spectrum of **1** in C_6D_6. (Insert shows portion of HSQC spectrum).

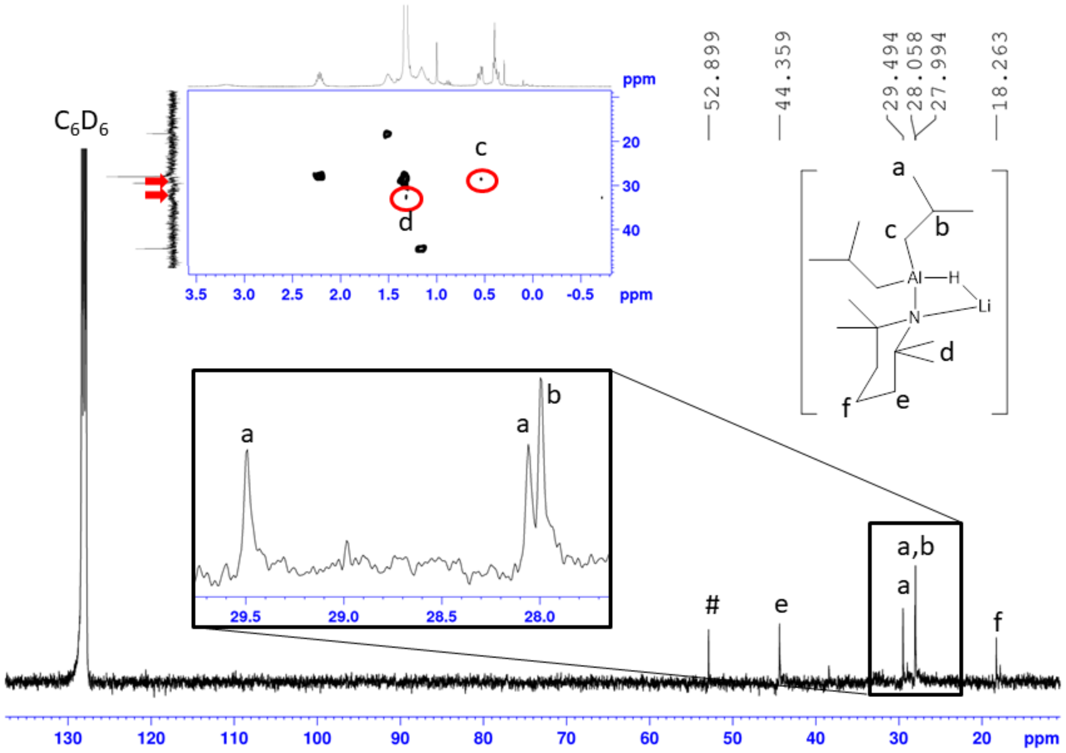

Figure S3: ^{7}Li NMR spectrum of **1** in C_6D_6.

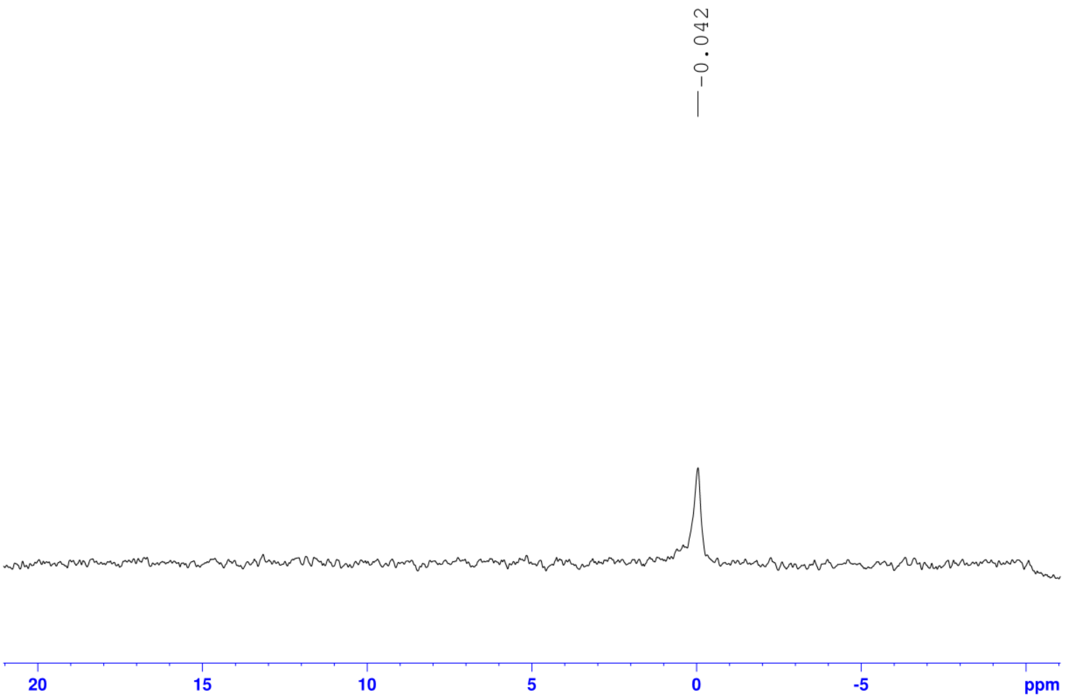

Figure S4: DOSY spectrum of **1** in C_6D_6 (TMS as internal reference).

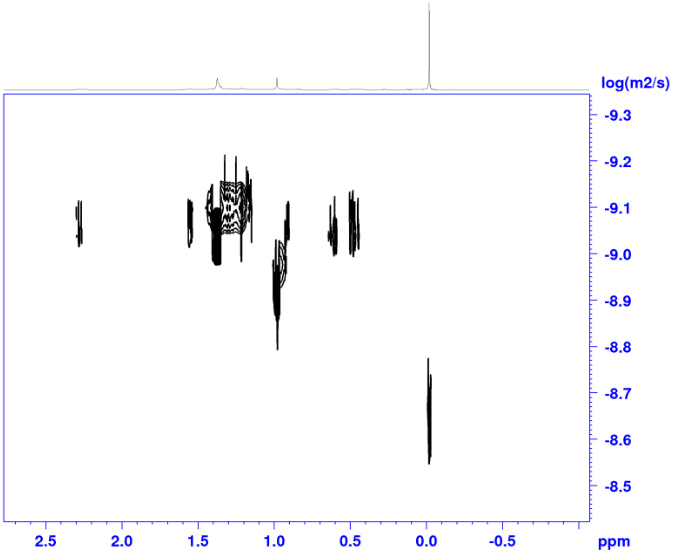

Compound	D [m^2 s^{-1}]	MW_{calc} [g mol^{-1}]	MW_{est} [g mol^{-1}]	Error [%]
[LiHAlTMP(*i*Bu)$_2$]$_2$	7.088E^{-10}	579	524	13

The diffusion coefficient of **1** indicates a molecular weight of 524 g mol^{-1}. This result suggests a dimer [LiHAlTMP(*i*Bu)$_2$]$_2$ in solution.

Preparation of [(THF)$_3$·LiHAlTMP(iBu)$_2$] (2): To a solution of [LiHAlTMP(*i*Bu)$_2$] (145 mg, 0.25 mmol) in toluene (4 mL), THF (0.10 mL, 1.5 mmol) was added, and a clear solution was obtained. The reaction solution was stirred for 30 min, and 6 mL of *n*-hexane was added. The reaction solution was left in the freezer at −26°C for 24 h. [(THF·)$_3$LiHAlTMP(*i*Bu)$_2$] (**2**) precipitated from the solution as colourless crystals and was isolated in 60.5% yield (153 mg, 0.30 mmol).

^{1}H NMR (400.13 Hz, C_6D_6, 298 K) δ 3.83 (br. s, 1H, Li-*H*-Al), 3.41 (m, 12H, C*H*$_2$(THF)), 2.38 (sept, 2H, AlCH$_2$C*H*(CH$_3$)$_2$), 1.62 (m, 2H, *p*-C*H*$_2$(TMP)), 1.45 (s, 12H, 4 x C*H*$_3$(TMP)), 1,43 (d, 12H, $^3J_{HH}$ = 6.5, AlCH$_2$CH(C*H*$_3$)$_2$), 1.28 (m, 12H, C*H*$_2$(THF)), 1.24 (m, 4H, *m*-C*H*$_2$(TMP)), 0.61 (dd, 2H, AlC*H*$_2$CH(CH$_3$)$_2$), 0.48 (dd, 2H, AlC*H*$_2$CH(CH$_3$)$_2$) ppm.

^{13}C NMR (100.61 Hz, C_6D_6, 298 K) δ 68.3 (*C*H$_2$(THF)), 52.8, (quaternary C TMP), 45.0 (*m*-*C*H$_2$(TMP)), 32.7 (*C*H$_3$(TMP)), 29.6 (Al*C*H$_2$CH(CH$_3$)$_2$), 28.4 (AlCH$_2$CH(*C*H$_3$)$_2$), 28.2 (AlCH$_2$CH(*C*H$_3$)$_2$), 25.4 (*C*H$_2$(THF)), 18.6 (*p*-*C*H$_2$(TMP)) ppm.

^{7}Li NMR (155.47 Hz, C_6D_6, 298 K) δ −0.83 (s, *Li*-H-Al) ppm.

Elemental analysis: *Calculated:* C: 68.87 %, H: 12.16 %, N: 2.77 %. *Found:* C: 69.14 %, H: 11.98 %, N: 2.34 %.

Figure S5: ^{1}H NMR spectrum of **2** in C_6D_6.

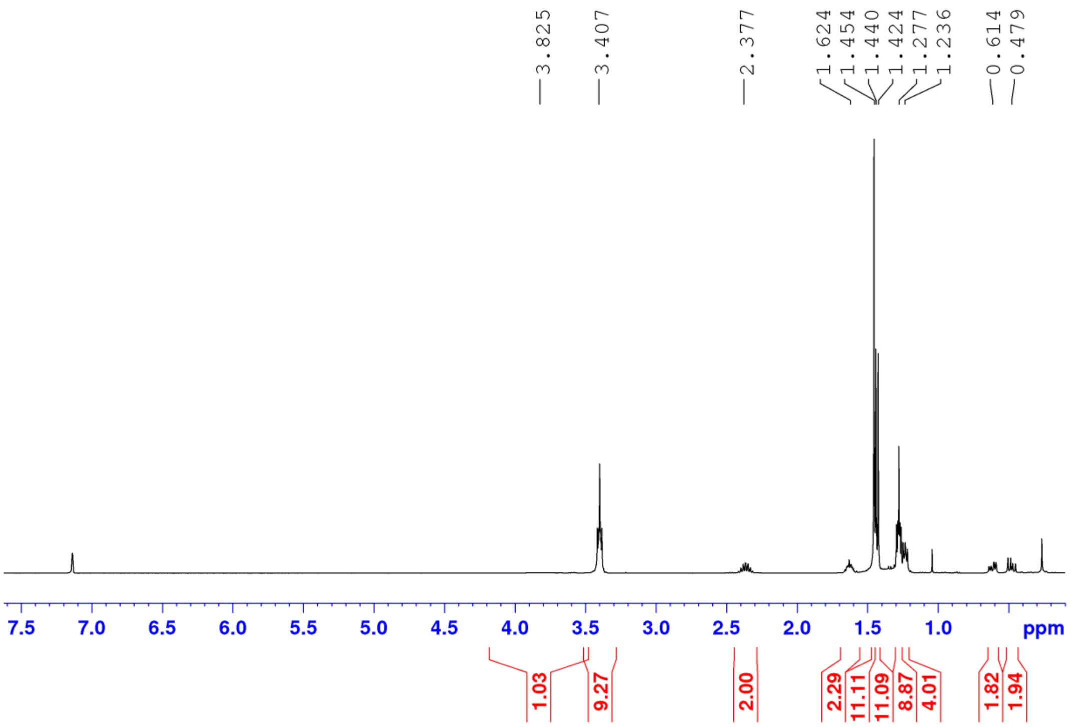

Figure S6: ^{13}C NMR spectrum of **2** in C_6D_6.

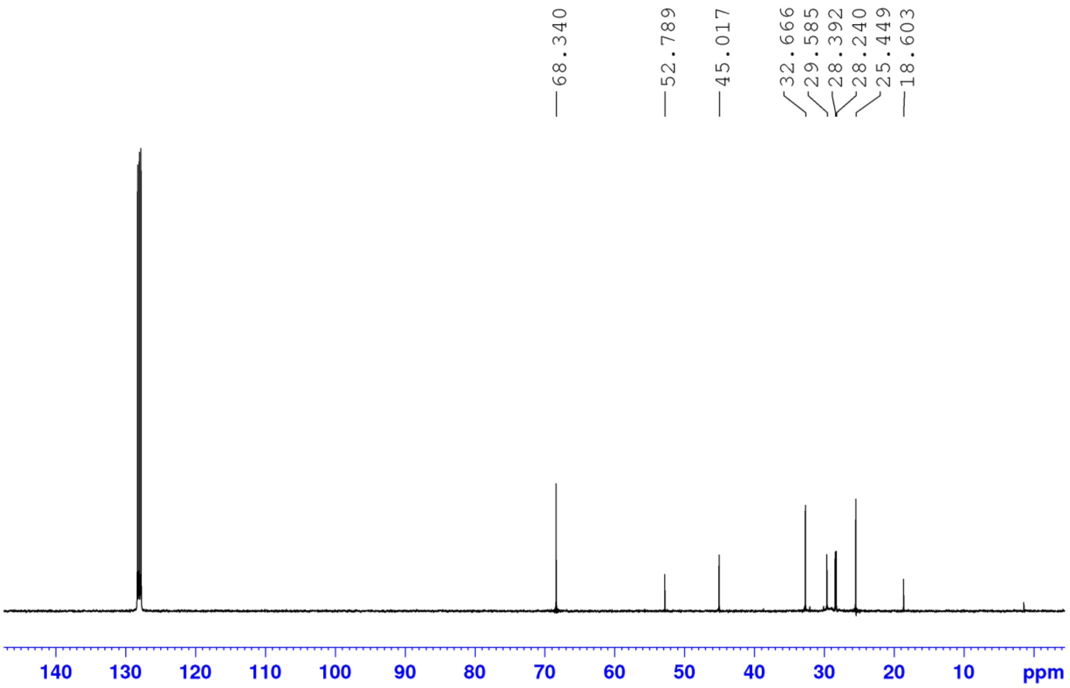

Figure S7: ^{7}Li NMR spectrum of **2** in C_6D_6.

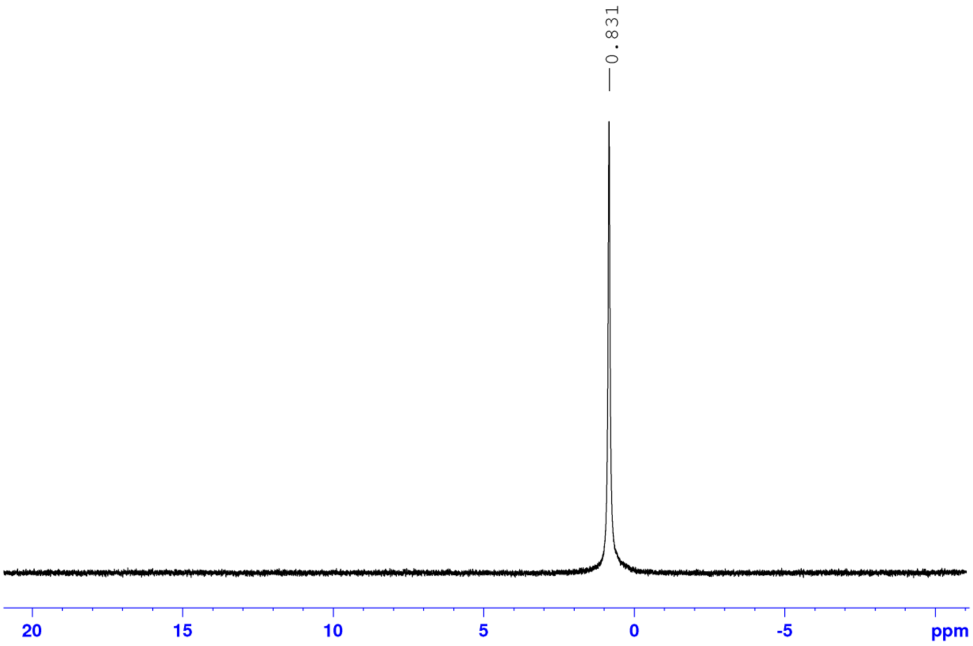

Figure S8: DOSY spectrum of **2** in C_6D_6 (tetraphenylnaphthalene as internal reference).

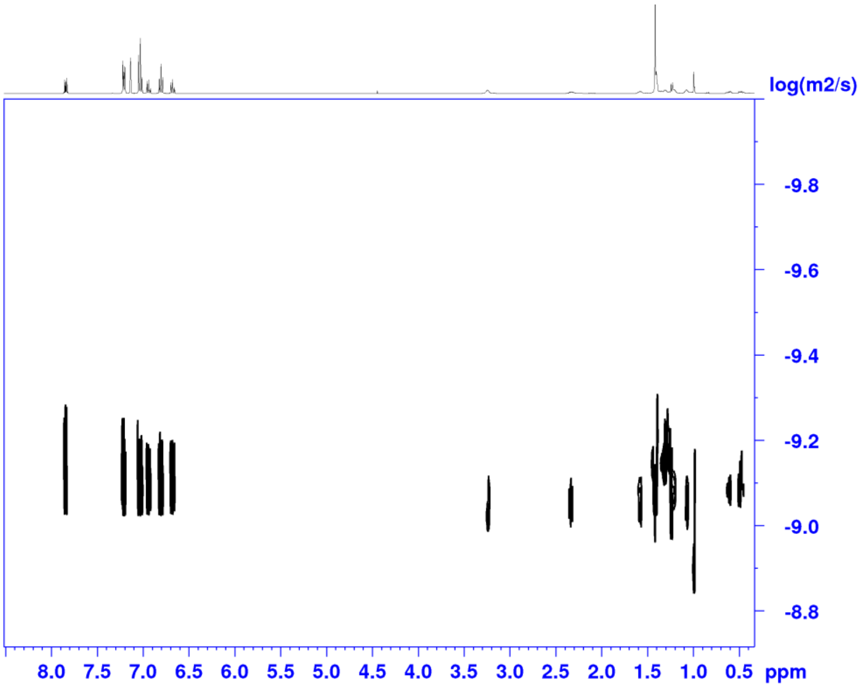

Compound	D [m^2 s^{-1}]	MW_{calc} [g mol^{-1}]	MW_{est} [g mol^{-1}]	Error [%]
[$(THF)_3$·LiHAlTMP(*i*Bu)$_2$]	7.515e^{-10}	506<br>444 (THF resonances)	486	4

The diffusion coefficient of **2** indicates a molecular weight of 506 g mol^{-1}. This result suggests the solid state structure is largely the same in solution, albeith with some THF desolvation.

Preparation of [PMDETA·LiHAlTMP(iBu)$_2$] (3): To a solution of [LiHAlTMP(*i*Bu)$_2$]$_2$ (145 mg, 0.25 mmol) in toluene (4 mL), PMDETA (0.10 mL, 0.5 mmol) was added, and a clear solution appeared. The reaction solution was stirred for 30 min, and afterwards 6 mL of *n*-hexane was added. The reaction solution was left in the freezer at −26°C for 24 h. [PMDETA·LiHAlTMP(*i*Bu)$_2$] (**3**) precipitated from the solution as colourless crystals and was isolated in 67% yield (153 mg, 0.33 mmol).

^{1}H NMR (400.13 Hz, C_6D_6, 298 K) δ 3.20 (br. s, 1H, Li-*H*-Al), 2.29 (sept, 2H, $^3J_{HH}$ = 6.5Hz, AlCH$_2$C*H*(CH$_3$)$_2$), 1.97 (br. s, 2H, *p*-C*H*$_2$(TMP)), 1.95 (s, 3H, C*H*$_3$(PMDETA)), 1.90 (s, 12H, 4 x C*H*$_3$(PMDETA)), 1.74 (m, 4H, *m*-C*H*$_2$(TMP)), 1.71 (s, 12H, 4 x C*H*$_3$(TMP)), 1.58 (br. s, C*H*$_2$(PMDTEA)), 1,46 (d, 6H, $^3J_{HH}$ = 6.5, AlCH$_2$CH(C*H*$_3$)$_2$), 1,45 (d, 6H, $^3J_{HH}$ = 6.5, AlCH$_2$CH(C*H*$_3$)$_2$), 0.40 (dd, 2H, $^3J_{HH}$ = 6.7 Hz, $^1J_{HH}$ = 13.2, AlC*H*$_2$CH(CH$_3$)$_2$), 0.31 (dd, 2H, $^3J_{HH}$ = 6.5 Hz, $^3J_{HH}$ = 13.2 Hz, AlC*H*$_2$CH(CH$_3$)$_2$) ppm.

^{13}C NMR (100.61 Hz, C_6D_6, 298 K) δ 57.1 (*C*H$_2$(PMDETA)), 53.2 (*C*H$_2$(PMDETA)), 45.7 (*C*H$_3$(PMDETA)), 45.0 (*C*H$_3$(PMDETA)), 43.9 (*m*-*C*H$_2$(TMP)), 34.5 (*C*H$_3$(TMP)), 30.7 (br, Al*C*H$_2$CH(CH$_3$)$_2$), 29.8 (AlCH$_2$CH(*C*H$_3$)$_2$), 28.7 (AlCH$_2$*C*H(CH$_3$)$_2$), 19.9 (*p*-*C*H$_2$(TMP)) ppm.

^{7}Li NMR (155.47 Hz, C_6D_6, 298 K) δ −0.38 (d, *Li*-H-Al) ppm.

Elemental analysis: *Calculated:* C: 67.49 %, H: 13.07 %, N: 12.11 %. *Found:* C: 67.36 %, H: 13.15 %, N: 12.01 %.

Figure S9: ^{1}H NMR spectrum of **3** in C_6D_6.

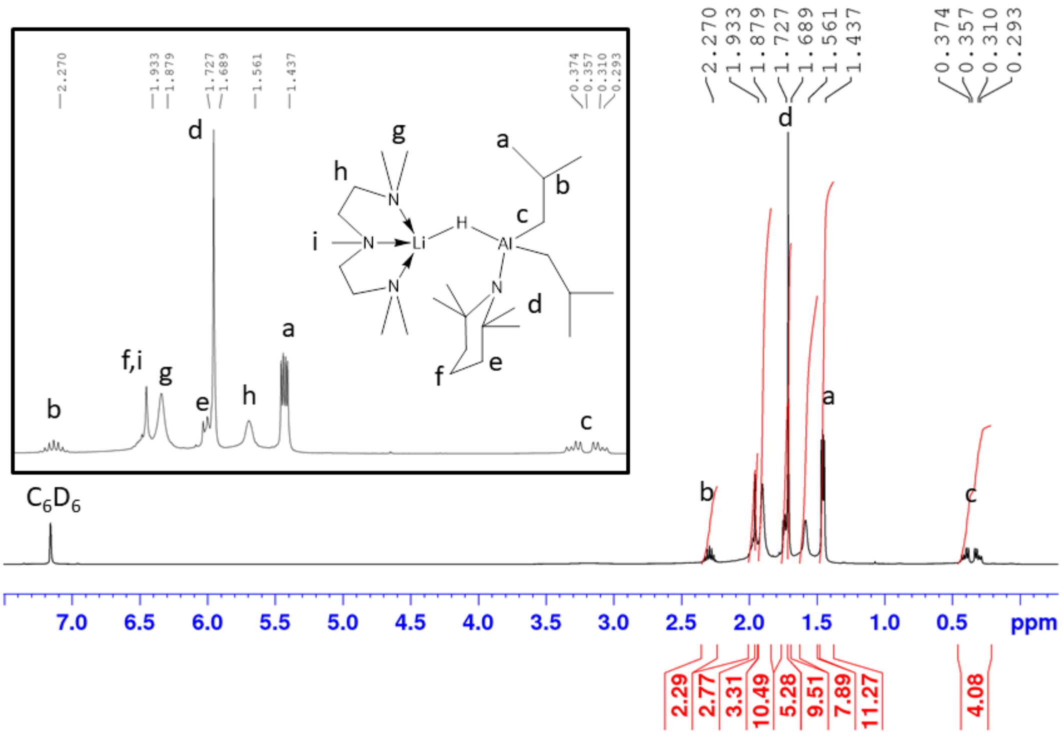

Figure S10: ^{13}C NMR spectrum of **3** in C_6D_6.

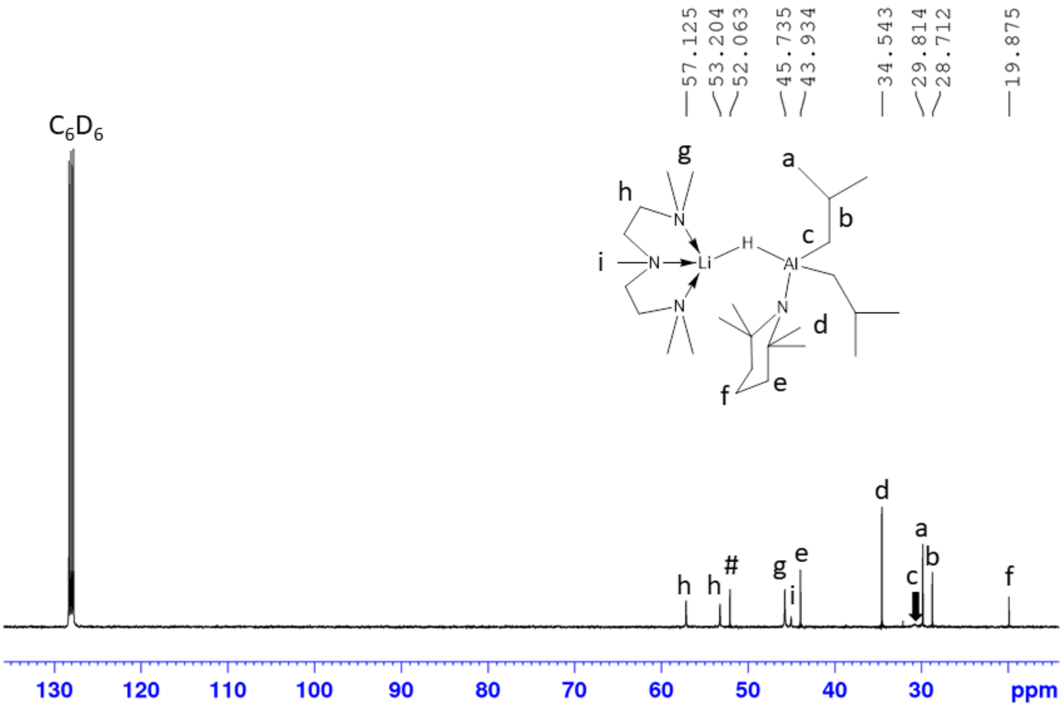

Figure S11: ^{7}Li NMR spectrum of **3** in C_6D_6.

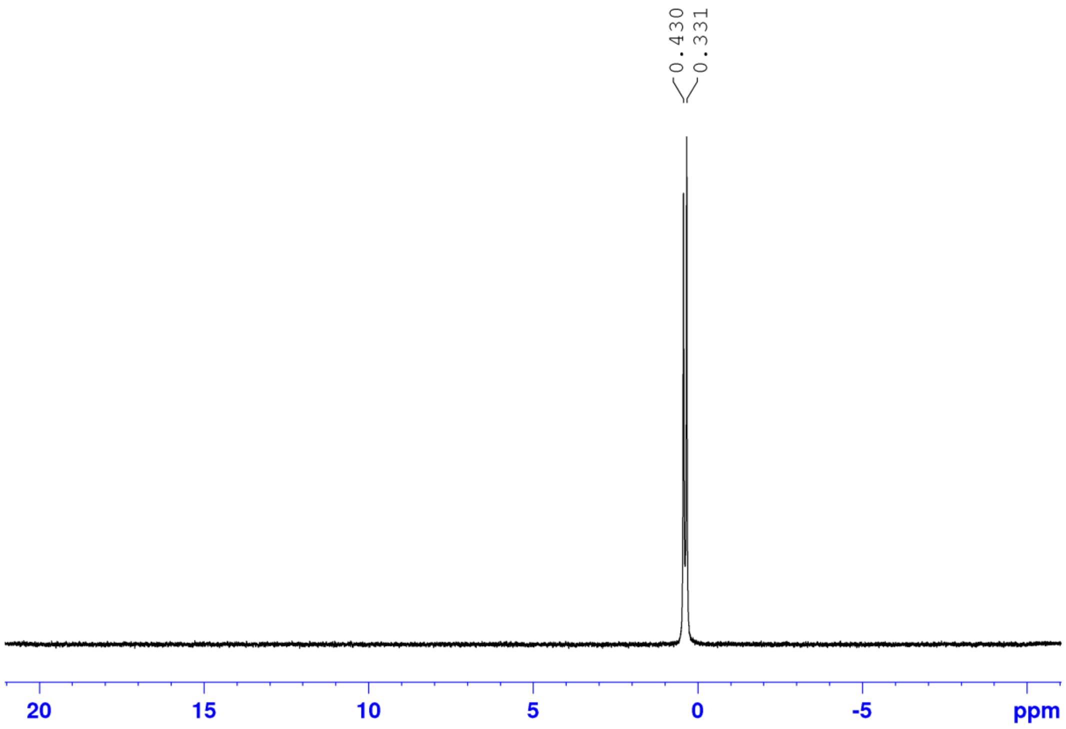

Figure S12: DOSY spectrum of **3** in C_6D_6 (TMS as internal standard).

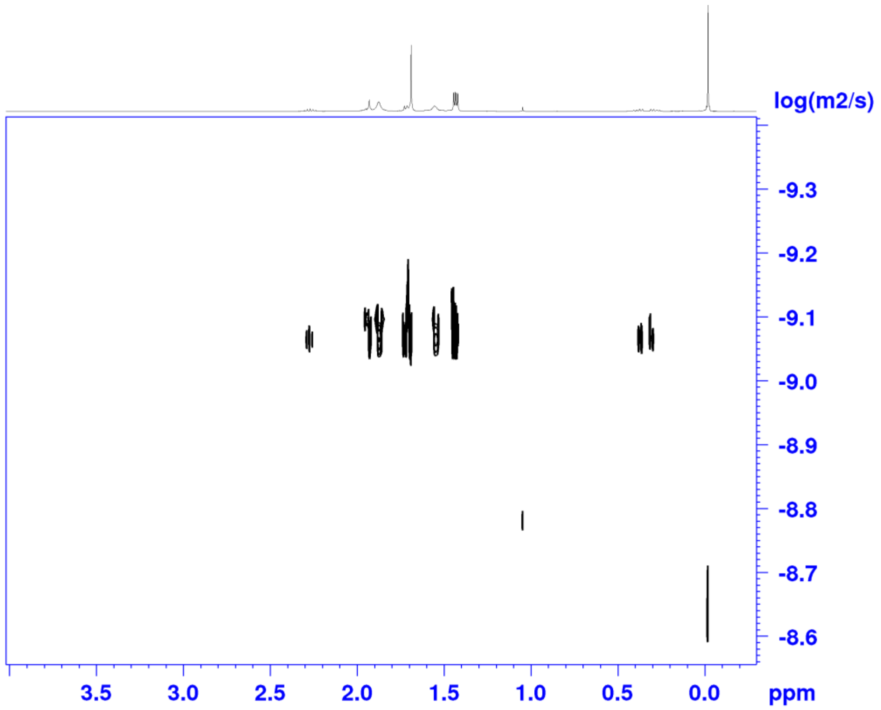

Compound	D [m^2 s^{-1}]	MW_{calc} [g mol^{-1}]	MW_{est} [g mol^{-1}]	Error [%]
[(PMDETA)LiHAlTMP(*i*Bu)$_2$]	7.427e^{-10}	446	463	4

The diffusion coefficient of [PMDETA·LiHAlTMP(*i*Bu)$_2$] indicates a molecular weight of 446 g mol^{-1}. This result is consistent with the calculated molecular weight of 463 g mol^{-1} (error:4%).

Preparation of [(Diglyme)$_2$·Li][Li(HAlTMP{*i*Bu}$_2$)] (4): To a solution of [LiHAlTMP(*i*Bu)$_2$]$_2$ (290 mg, 0.5 mmol) in toluene (6 mL), Diethylene glycol dimethyl ether (0.14 mL, 1 mmol) was added, and the reaction solution was stirred for 30 min. Afterwards 8 mL of *n*-hexane was added, and the reaction solution was left in the freezer at −26°C for 24 h. [(Diglyme)$_2$·Li][Li(HAlTMP{*i*Bu}$_2$)] (**4**) precipitated from the solution as colourless crystals and was isolated in 92% yield (390 mg, 0.46 mmol).

^{1}H NMR (400.13 Hz, C_6D_6, 298 K) δ 3.61 (br. s, 1H, Li-*H*-Al), 3.03 (br. t, 8H, $^3J_{HH}$ = 4.7 Hz, 4 x CH_3OCH_2), 3.00 (s, 12H, 4 x CH_3OCH_2), 2.84 (br. t, 8H, $^3J_{HH}$ = 4.7 Hz, 4 x CH_3OCH_2), 2.42 (sept, 4H, $^3J_{HH}$ = 6.5Hz, 4 x $AlCH_2CH(CH_3)_2$), 1.84 (br. s, 4H, 2 x *p*-CH_2(TMP)), 1.65 (s, 24H, 8 x CH_3(TMP)), 1.53 (m, 8H, 2 x *m*-CH_2(TMP)), 1,49 (d, 24H, $^3J_{HH}$ = 6.5, 4 x $AlCH_2CH(CH_3)_2$), 0.62 (dd, 4H, $^3J_{HH}$ = 6.0 Hz, $^1J_{HH}$ = 13.5, 2 x $AlCH_2CH(CH_3)_2$), 0.54 (dd, 2H, $^3J_{HH}$ = 7.7 Hz, $^3J_{HH}$ = 13.5 Hz, $AlCH_2CH(CH_3)_2$) ppm.

^{13}C NMR (100.61 Hz, C_6D_6, 298 K) δ 70.1 (CH_3OCH_2), 68.5 (CH_3OCH_2), 59.1 (CH_3OCH_2), 44.2 (*m*-CH_2(TMP)), 33.6 (CH_3(TMP)), 30.0 (just observable in the HSQC, $AlCH_2CH(CH_3)_2$), 29.9 ($AlCH_2CH(CH_3)_2$), 28.9 ($AlCH_2CH(CH_3)_2$), 28.5 ($AlCH_2CH(CH_3)_2$), 19.3 (*p*-CH_2(TMP)) ppm.

^{7}Li NMR (155.47 Hz, C_6D_6, 298 K) δ −0.32 (s, *Li*-H-Al) ppm.

^{7}Li NMR (155.47 Hz, C_6D_6, 203 K) δ −1.96 (s, *Li*-H-Al), −0.36 (s, *Li*-H-Al), 1.68 ppm.

Elemental analysis: *Calculated:* C: 65.30 %, H: 12.03 %, N: 3.31 %. *Found:* C: 64.77 %, H: 12.40 %, N: 3.31 %.

Figure S13: ^{1}H NMR spectrum of **4** in C_6D_6.

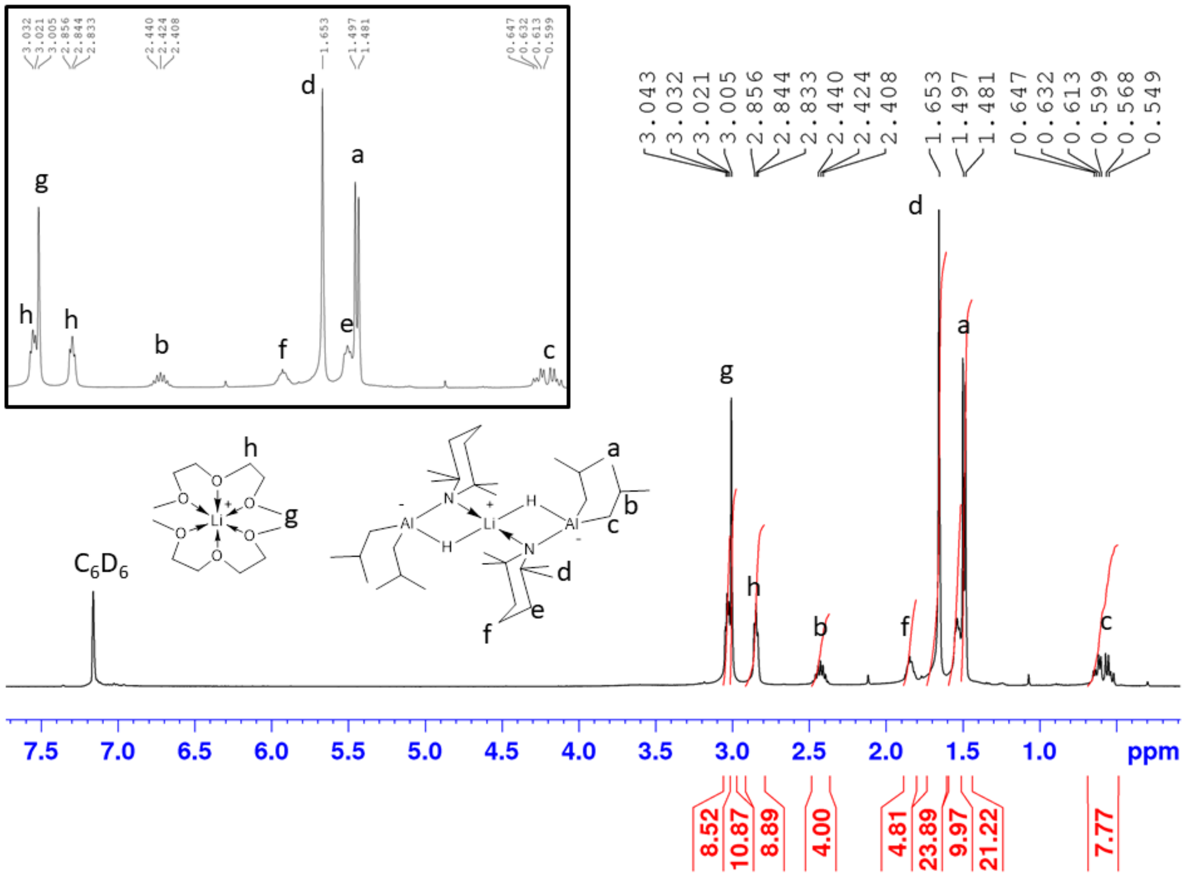

Figure S14: ^{13}C NMR spectrum of **4** in C_6D_6.

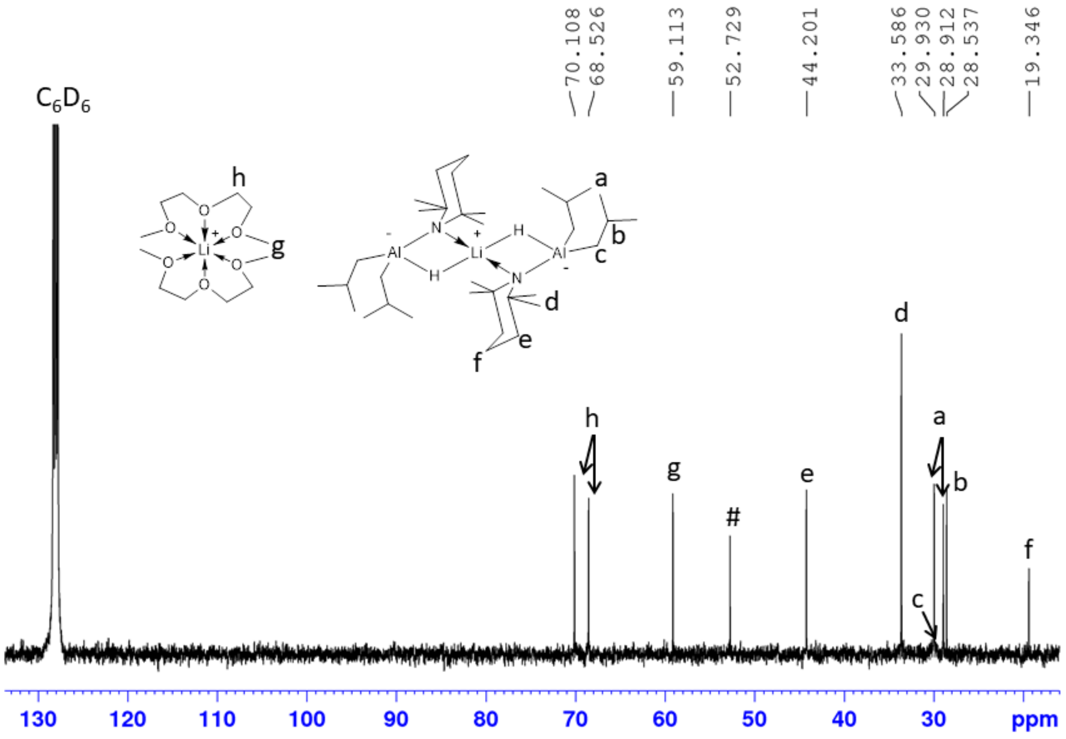

Figure S15: Variable temperature 7Li study of **4** in d_8-toluene.

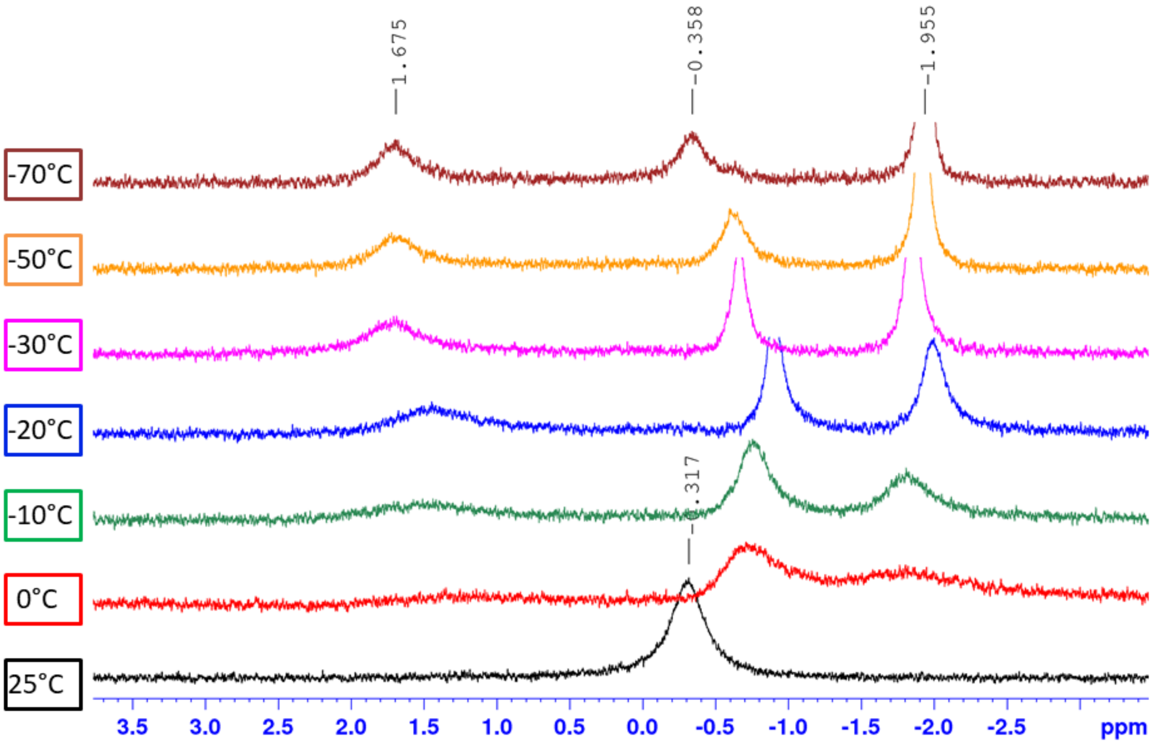

Figure S16: DOSY spectrum of **4** in C_6D_6 (TMS as internal standard).

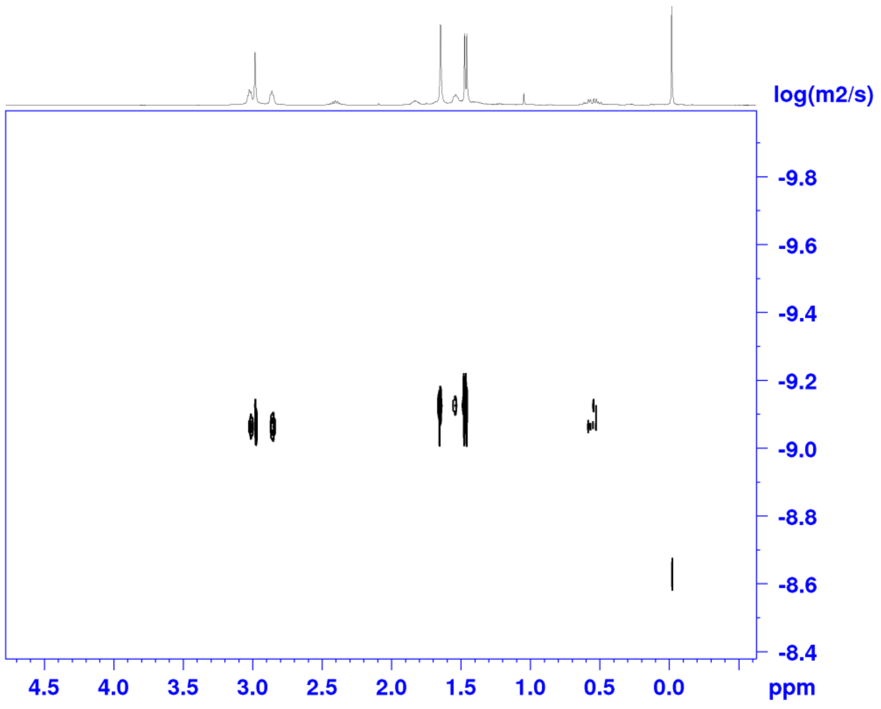

Compound	D [m^2 s^{-1}]	MW_{calc} [g mol^{-1}]	MW_{est} [g mol^{-1}]	Error [%]
[(Diglyme)$_2$·Li][Li(HAlTMP{*i*Bu}$_2$)]	5.546e^{-11}	846<br>846	509 diglyme resonances	40<br>27

			615 *i*Bu_2/TMP resonances	

Here the diffusion coefficient of the diglyme resonances is markedly different than those of the remainder of the resonances in the spectrum. The estimated molecular weight values are also different and are not in accordance with the value of the structure in the crystal structure. They are also significantly different than the expected value if **4** resembled the structure of **3** in solution (423.59 g mol^{-1}). That the values lie intermediate between the two structure extremes, we suggest that in solution ligand exchange is occurring giving the experimentally observed values.

Preparation of [DABCO·{LiHAlTMP(iBu)$_2$}$_2$] (5): To a solution of [LiHAlTMP(*i*Bu)$_2$] (290 mg, 0.50 mmol) in toluene (4 mL), DABCO (56 mg, 0.50 mmol) was added, and a clear solution was obtained. The reaction solution was stirred for 30 min, and 6 mL of *n*-hexane was added. The reaction solution was left in the freezer at −26°C for 24 h. [DABCO·{LiHAlTMP(iBu)$_2$}$_2$] (**5**) precipitated from the solution as colourless crystals and was isolated in 94% yield (324 mg, 0.47 mmol).

^{1}H NMR (400.13 Hz, C_6D_6, 298 K) δ 3.60 (br. s, 2H, Li-*H*-Al), 2.39 (sept, 4H, $^3J_{HH}$ = 6.5Hz, AlCH$_2$C*H*(CH$_3$)$_2$), 2.02 (s, 12H, C*H*$_2$(DABCO)), 1.64 (br. s, 4H, *p*-C*H*$_2$(TMP)), 1.47 (d, 24H, $^3J_{HH}$ = 6.5, AlCH$_2$CH(C*H*$_3$)$_2$), 1.42 (s, 24H, 4 x C*H*$_3$(TMP)), 1.19 (m, 8H, *m*-C*H*$_2$(TMP)), 0.70 (dd, 2H, $^3J_{HH}$ = 6.7 Hz, $^1J_{HH}$ = 13.2, AlC*H*$_2$CH(CH$_3$)$_2$), 0.66 (dd, 2H, $^3J_{HH}$ = 6.5 Hz, $^3J_{HH}$ = 13.2 Hz, AlC*H*$_2$CH(CH$_3$)$_2$) ppm.

^{13}C NMR (100.61 Hz, C_6D_6, 298 K) δ 46.0 (*C*H$_2$(DABCO)), 45.4 (*m*-*C*H$_2$(TMP)), 32.5 (*C*H$_3$(TMP)), 29.6 (br, Al*C*H$_2$CH(CH$_3$)$_2$), 29.6 (br, AlCH$_2$CH(*C*H$_3$)$_2$), 28.3 (AlCH$_2$CH(*C*H$_3$)$_2$), 28.3 (AlCH$_2$*C*H(CH$_3$)$_2$), 18.5 (*p*-*C*H$_2$(TMP)) ppm.

^{7}Li NMR (155.47 Hz, C_6D_6, 298 K) δ 1.06 (s, *Li*-H-Al) ppm.

Elemental analysis: *Calculated:* C: 69.53 %, H: 12.55 %, N: 8.11 %. *Found:* C: 69.55 %, H: 12.24 %, N: 8.03 %.

Figure S17: ^{1}H NMR spectrum of **5** in C_6D_6.

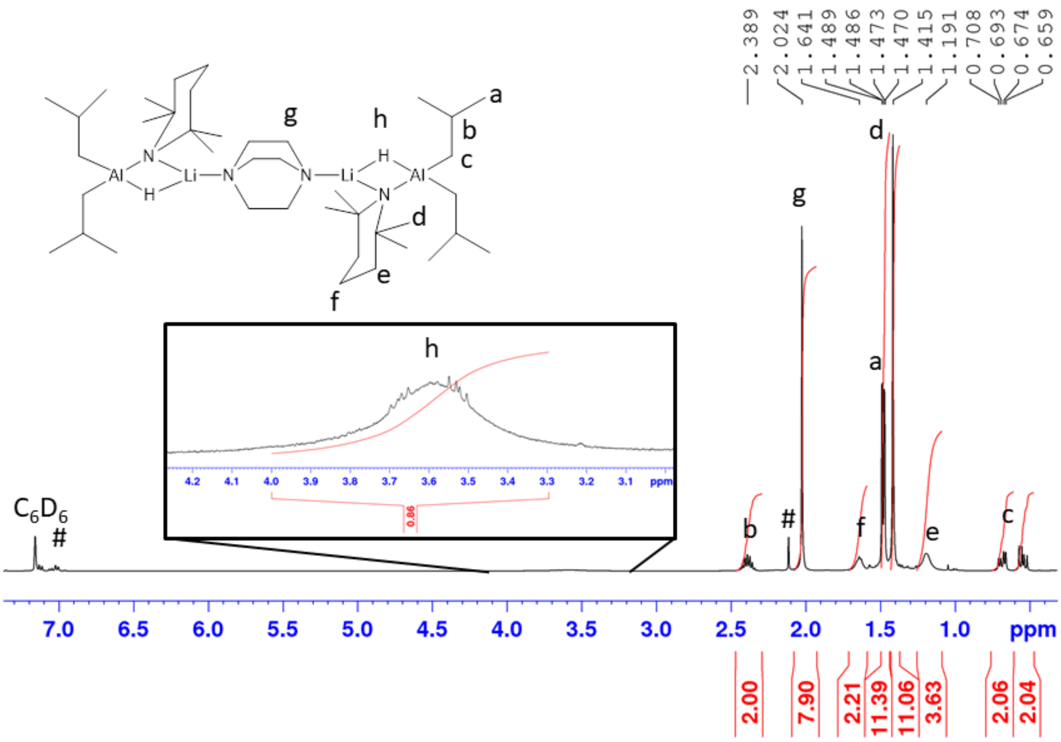

Figure S18: ^{13}C NMR spectrum of **5** in C_6D_6.

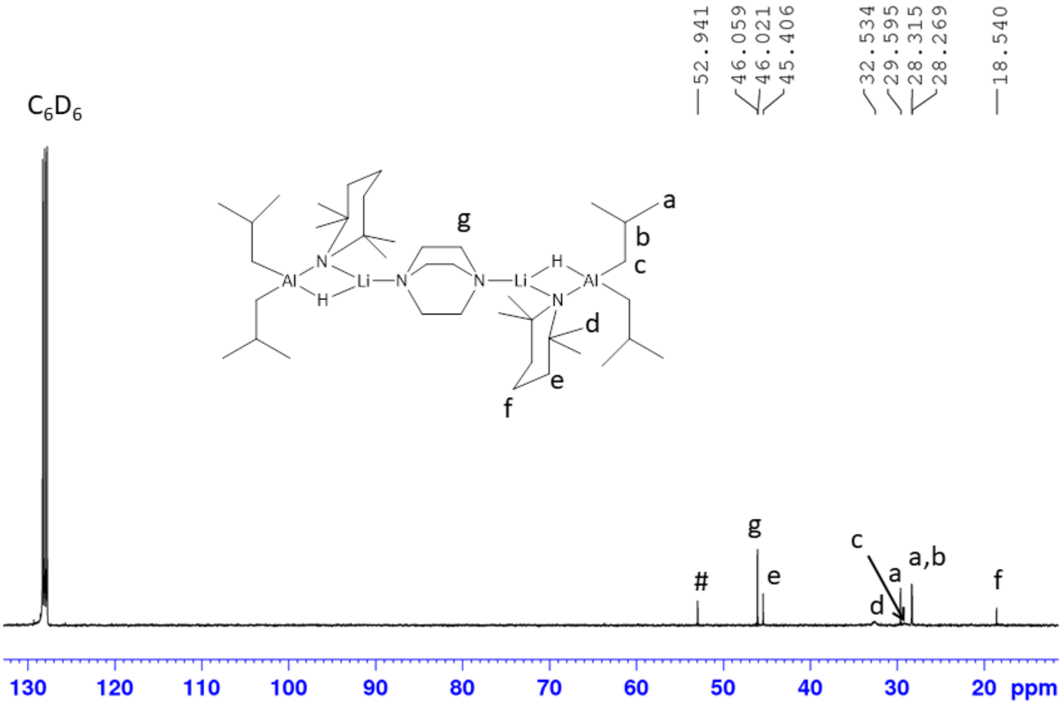

Figure S19: ^{7}Li NMR spectrum of **5** in C_6D_6.

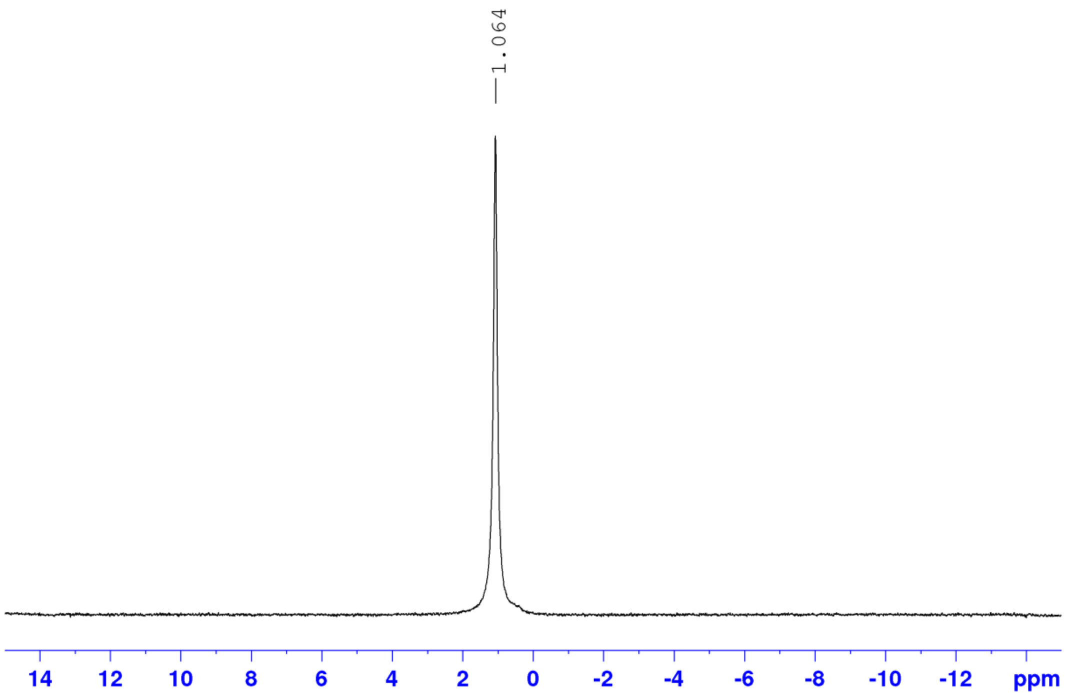

Figure S20: DOSY NMR spectrum of **5** in C_6D_6 (tetraphenylnaphthalene as internal standard).

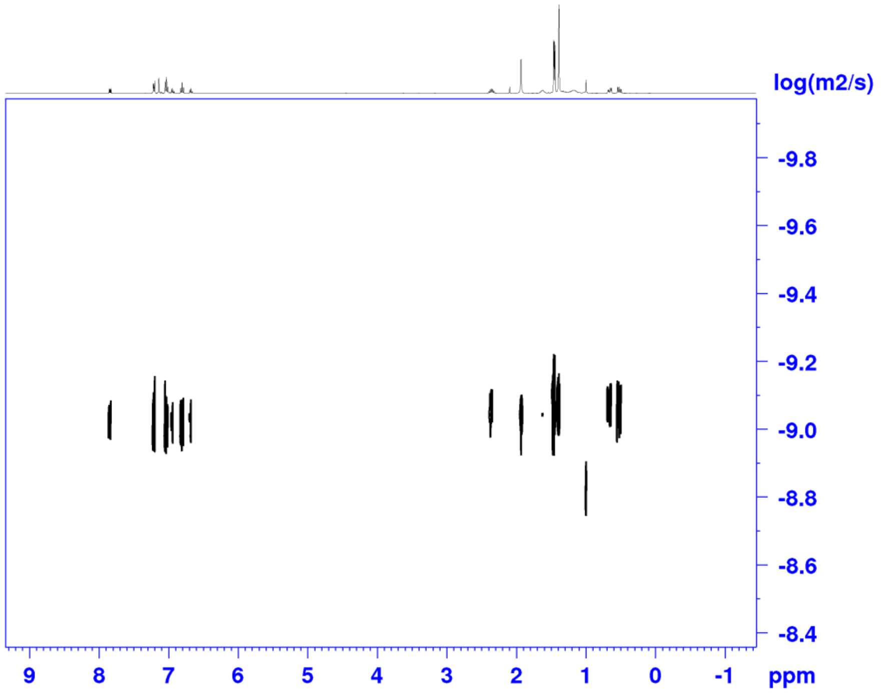

Compound	D [m^2 s^{-1}]	MW_{calc} [g mol^{-1}]	MW_{est} [g mol^{-1}]	Error [%]
[DABCO·{LiHAlTMP(iBu)$_2$}$_2$]	5.802e^{-11}	691	620	11

The estimatated molecular weight is below that of the expected mass therefore we suggest an equilibrium process between structures resembling **5** and **6**.

Preparation of [DABCO·LiHAlTMP(iBu)$_2$] (6): To a solution of [LiHAlTMP(*i*Bu)$_2$] (290 mg, 0.50 mmol) in toluene (4 mL), DABCO (112 mg, 1.0 mmol) was added, and a clear solution was obtained. The reaction solution was stirred for 30 min, and 6 mL of *n*-hexane was added. The reaction solution was left in the freezer at −26°C for 24 h. [DABCO·LiHAlTMP(iBu)$_2$] (**6**) precipitated from the solution as colourless crystals and was isolated in 38% yield (153 mg, 0.38 mmol).

^{1}H NMR (400.13 Hz, C_6D_6, 298 K) δ 3.60 (br. s, 1H, Li-*H*-Al), 2.39 (sept, 2H, AlCH$_2$C*H*(CH$_3$)$_2$), 2.07 (s, 12H, C*H*$_2$(DABCO)), 1.65 (br. s, 2H, *p*-C*H*$_2$(TMP)), 1.46 (d, 12H, AlCH$_2$CH(C*H*$_3$)$_2$), 1.41 (s, 12H, 4 x C*H*$_3$(TMP)), 1.19 (m, 4H, *m*-C*H*$_2$(TMP)), 0.69 (dd, 2H, $^3J_{HH}$ = 6.7 Hz, AlC*H*$_2$CH(CH$_3$)$_2$), 0.54 (dd, 2H, $^3J_{HH}$ = 6.5 Hz, AlC*H*$_2$CH(CH$_3$)$_2$) ppm.

^{13}C NMR (100.61 Hz, C_6D_6, 298 K) δ 46.3 (*C*H$_2$(DABCO)), 45.4 (*m*-*C*H$_2$(TMP)), 32.6 (*C*H$_3$(TMP)), 29.6 (Al*C*H$_2$CH(CH$_3$)$_2$), 29.6 (br, AlCH$_2$CH(*C*H$_3$)$_2$), 28.3 (AlCH$_2$CH(*C*H$_3$)$_2$), 28.3 (AlCH$_2$*C*H(CH$_3$)$_2$), 18.5 (*p*-*C*H$_2$(TMP)) ppm.

^{7}Li NMR (155.47 Hz, C_6D_6, 298 K) δ 1.08 (s, *Li*-H-Al) ppm.

Elemental analysis: *Calculated:* C: 68.79 %, H: 12.30 %, N: 10.46 %. *Found:* C: 69.27 %, H: 12.04 %, N: 10.09 %.

Figure S21: ^{1}H NMR spectrum of **6** in C_6D_6

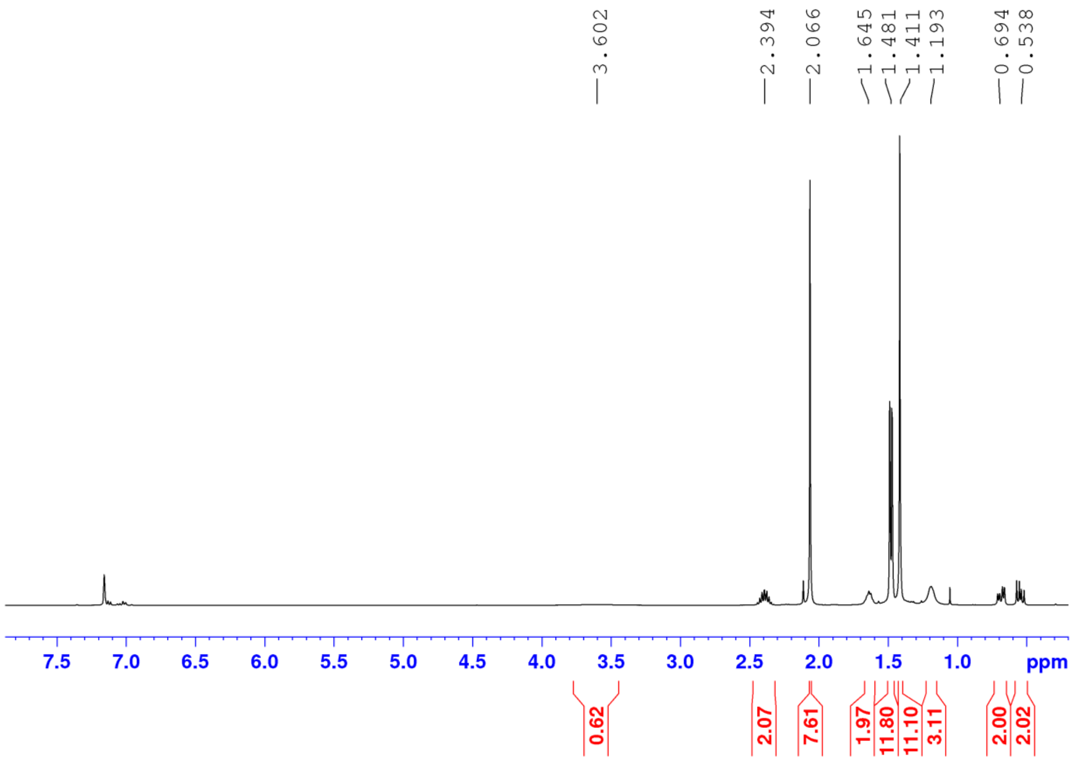

Figure S22: ^{13}C NMR spectrum of **6** in C_6D_6

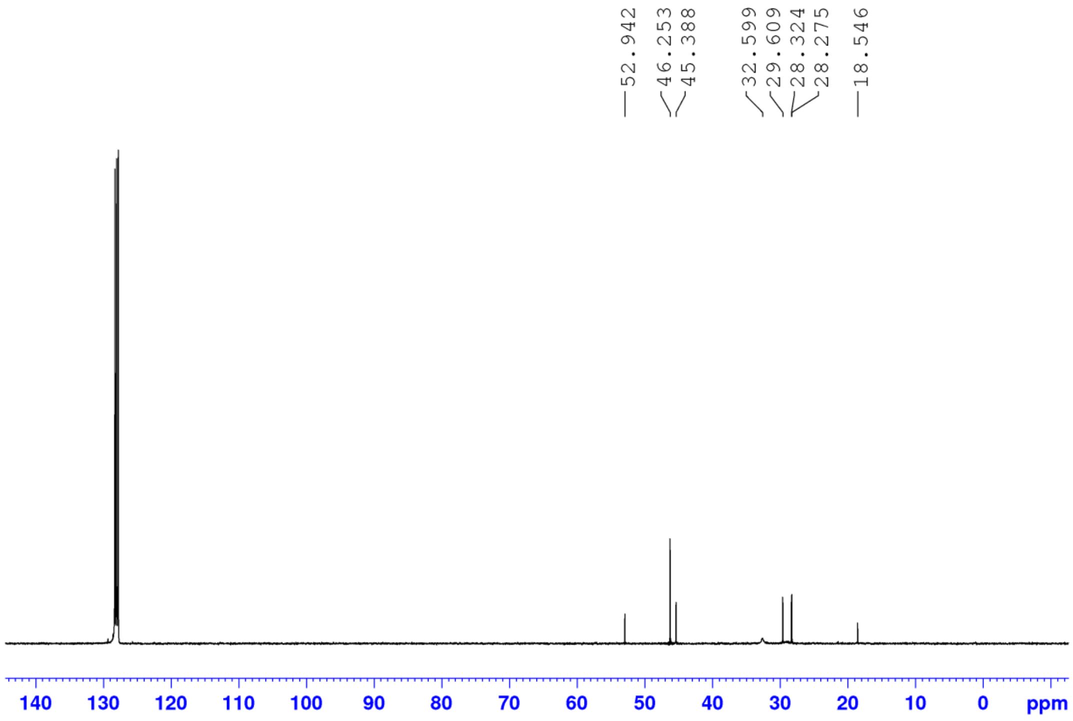

Figure S23: ^{7}Li NMR spectrum of **6** in C_6D_6

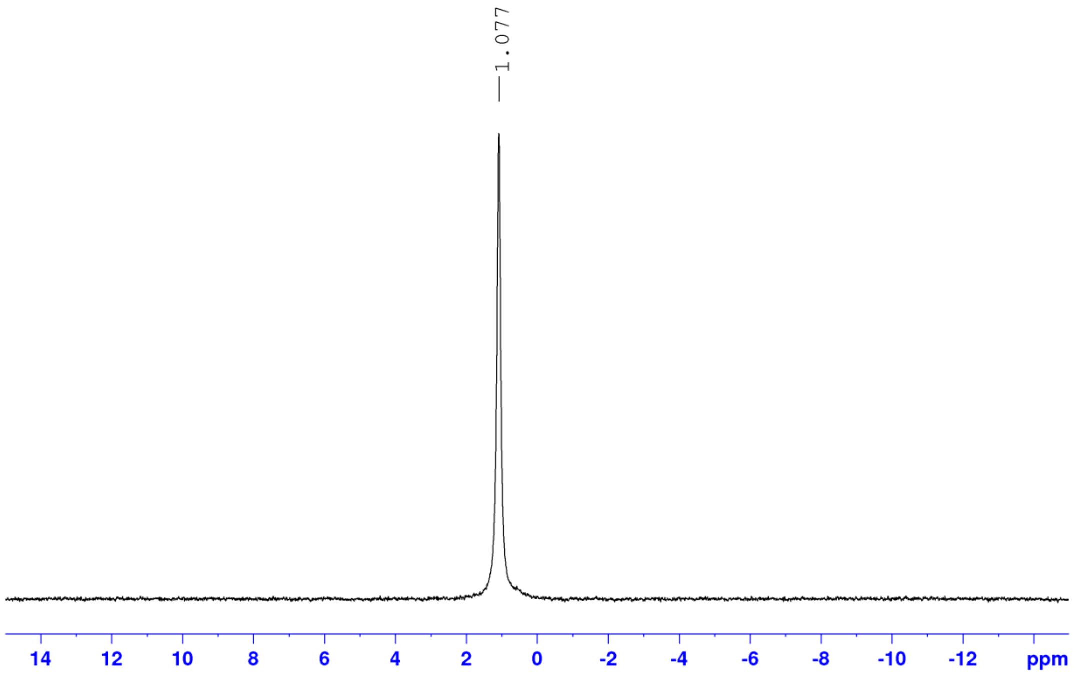

Figure S24: DOSY NMR spectrum of **6** in C_6D_6 (tetraphenylnaphthalene as internal standard).

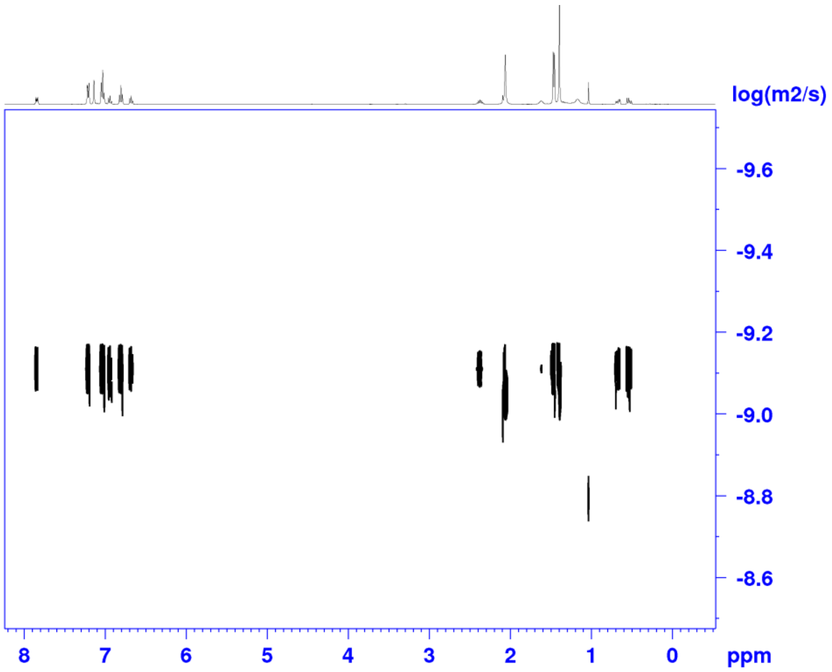

Compound	D [m^2 s^{-1}]	MW_{calc} [g mol^{-1}]	MW_{est} [g mol^{-1}]	Error [%]
[DABCO·{LiHAlTMP(iBu)$_2$}$_2$]	5.397 e^{-11}	402	514	22

The estimatated molecular weight is higher than that of the expected mass therefore we suggest an equilibrium process between structures resembling **5** and **6**, givent the similar spectroscopic characteristics.

Hydroboration catalysis

Catalytic reactions all performed at room temperature in C_6D_6 at room temperature, unless otherwise stated. Loadings are: **1** (2.5 mol%); **2** (5 mol%); **3** (5 mol%) **4** (2.5 mol%); **5** (2.5 mol%); **6** (5 mol%), unless otherwise stated.

Benzaldehyde

Figure S25: ^{1}H NMR spectra of benzaldehyde hydroboration using **1**, **3** or **4** in C_6D_6.

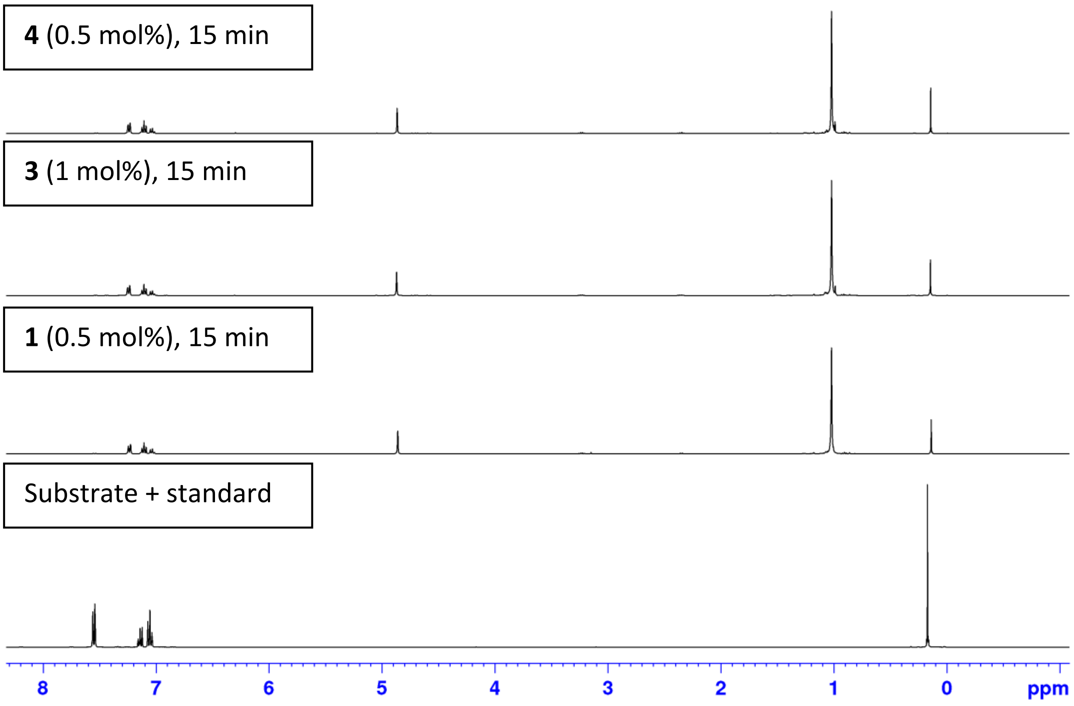

Figure S26: ^{11}B NMR spectrum of benzaldehyde hydroboration using **1** in C_6D_6.

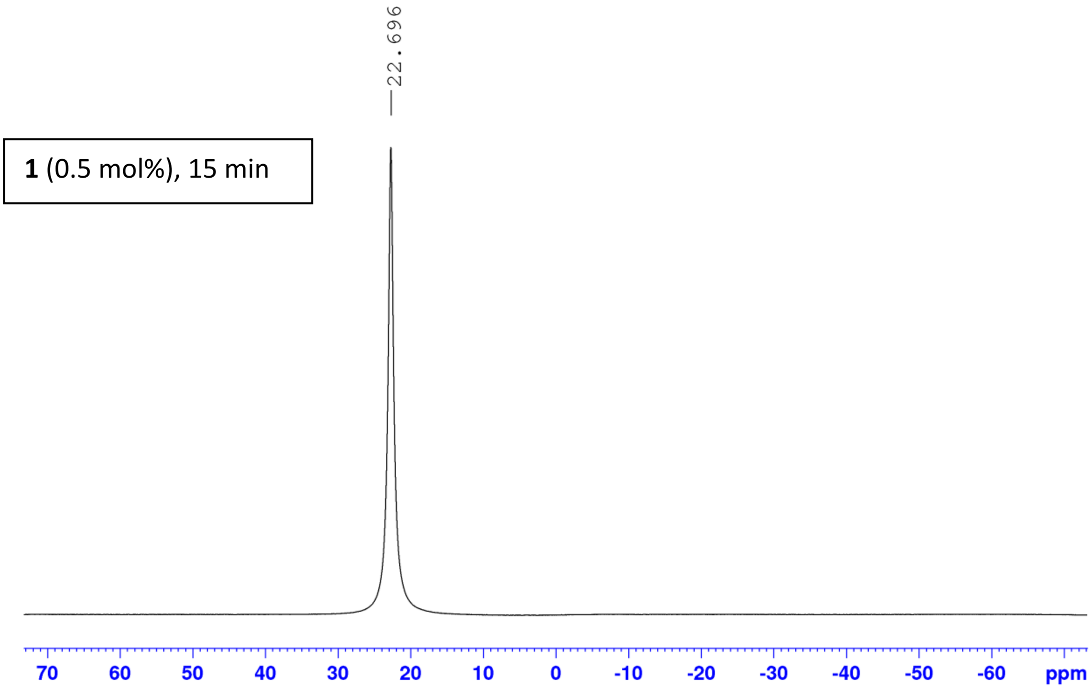

Cinnamaldehyde

Figure S27: ^{1}H NMR spectra of cinnamaldehyde hydroboration using **1**, **3** or **4** in C_6D_6.

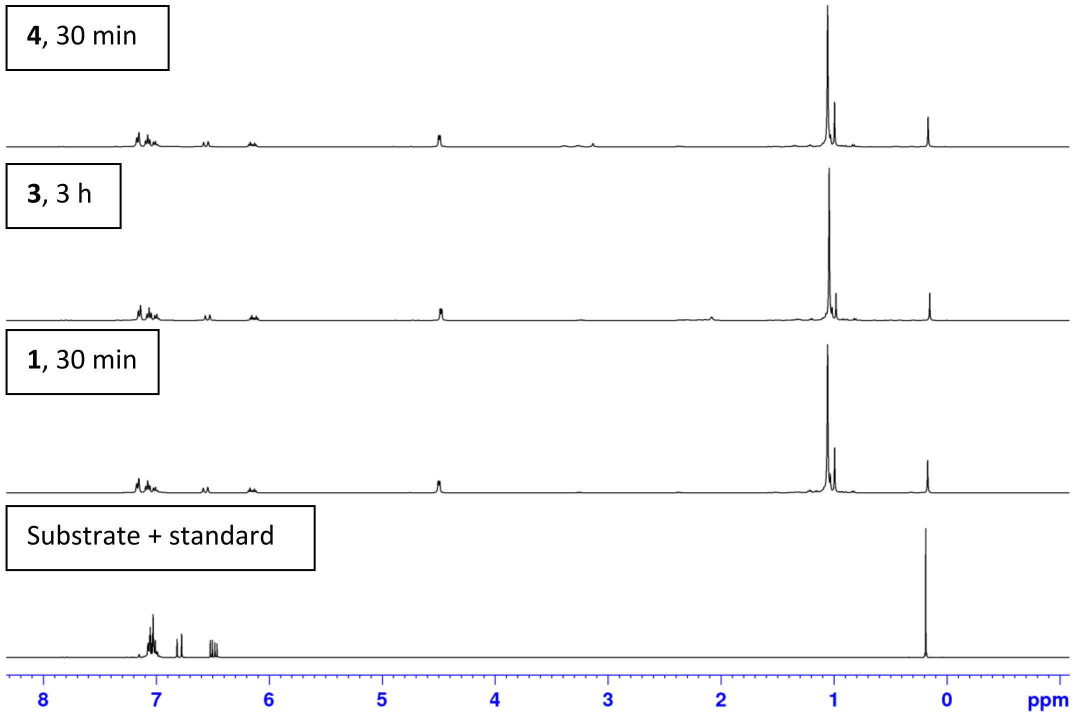

Figure S28: ^{11}B NMR spectrum of cinnamaldehyde hydroboration using **3** in C_6D_6.

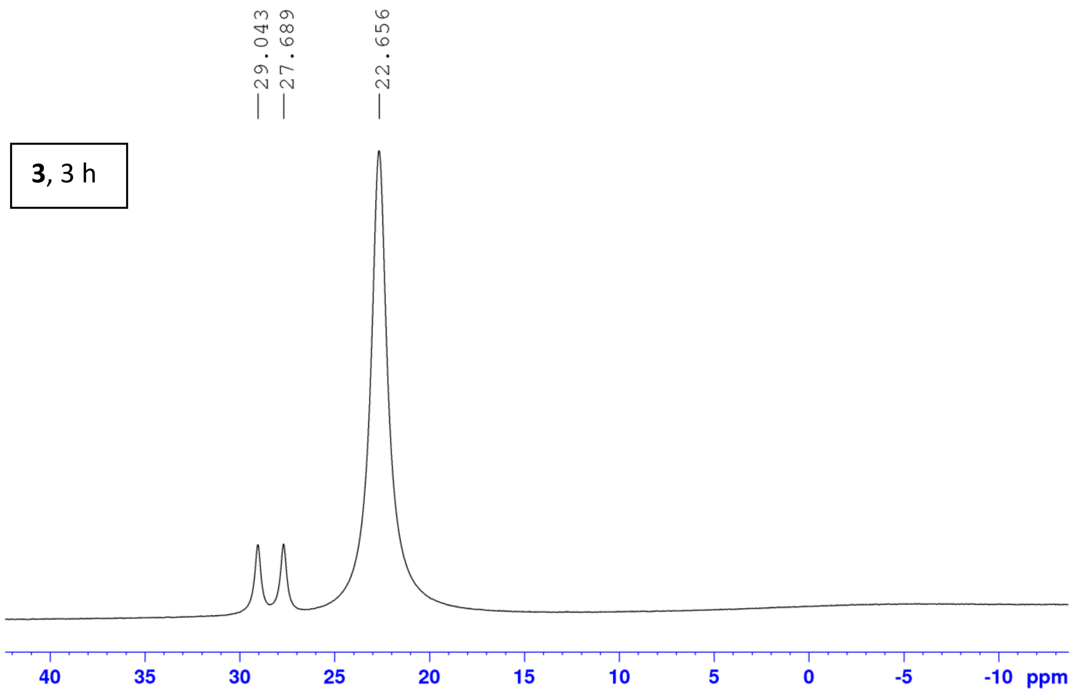

Figure S29: ^{1}H NMR spectra of cinnamaldehyde hydroboration using **2**, **5** or **7** in C_6D_6.

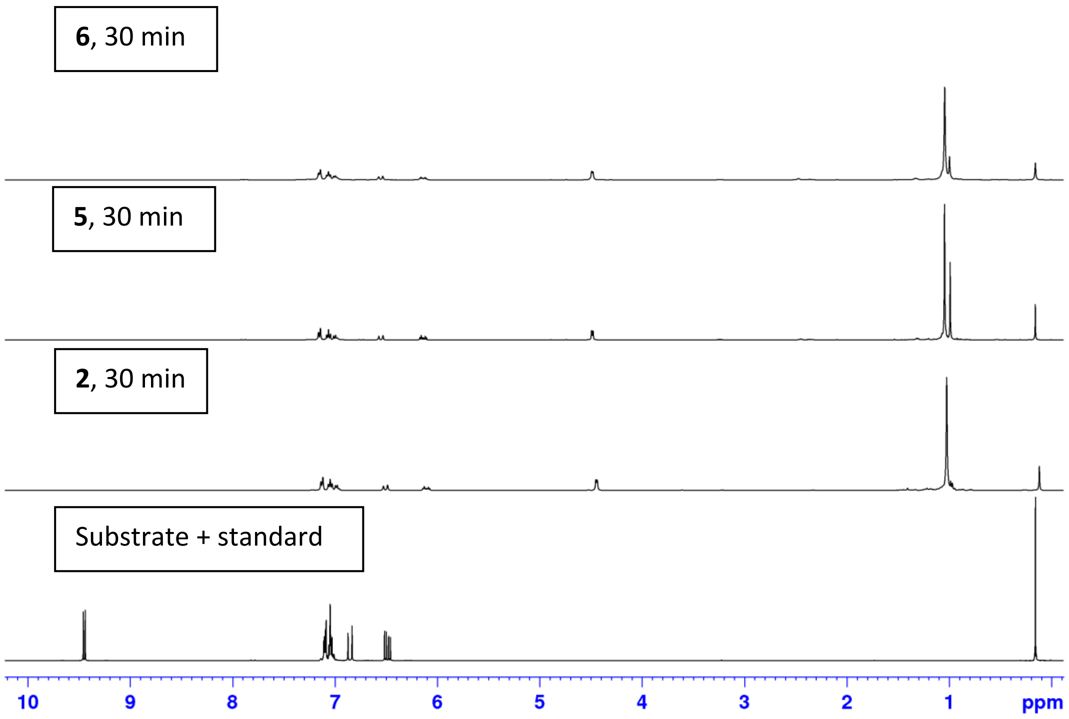

Acetophenone

Figure S30: ^{1}H NMR spectra of acetophenone hydroboration using **1** in C_6D_6.

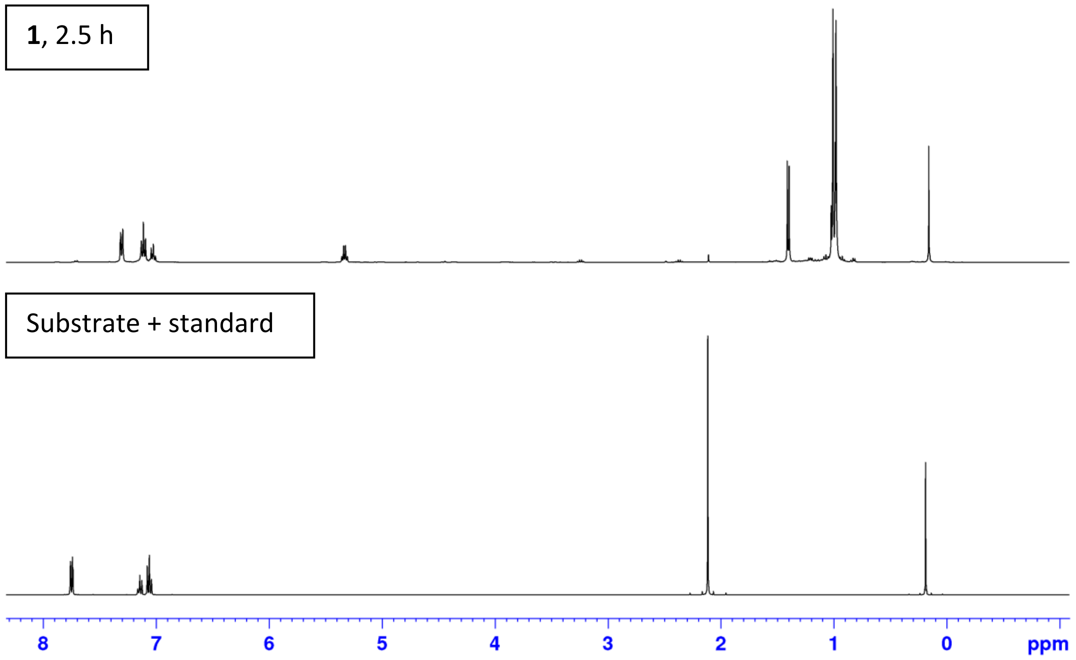

Figure S31: ^{1}H NMR spectra of acetophenone hydroboration using **3** in C_6D_6.

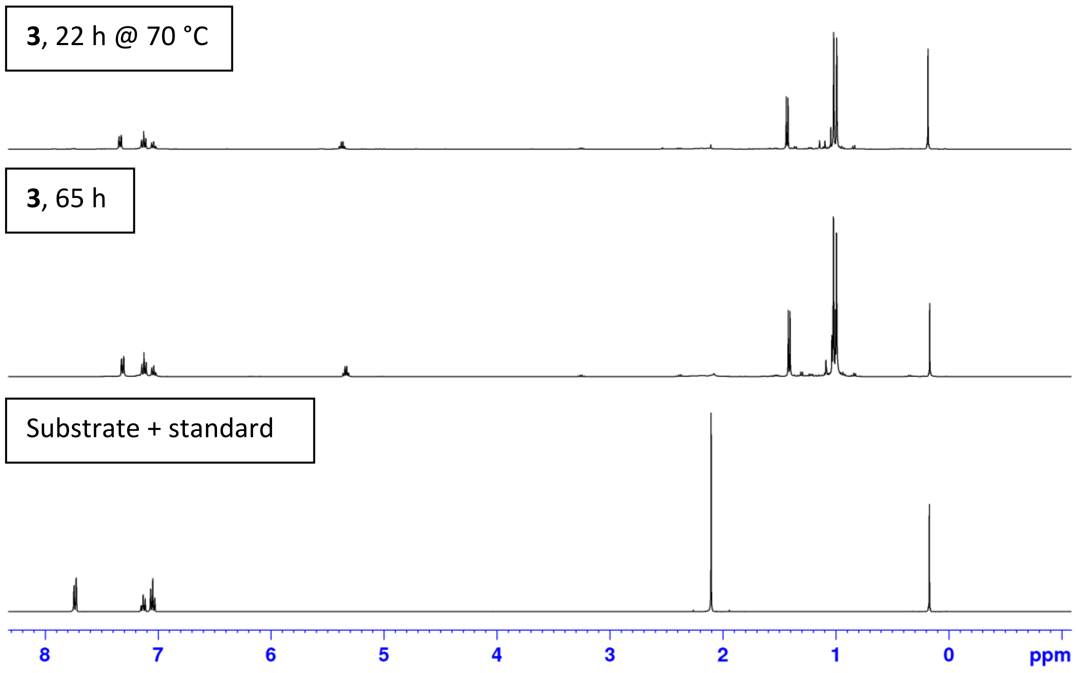

Figure S32: ^{11}B NMR spectrum of acetophenone hydroboration using **3** in C_6D_6.

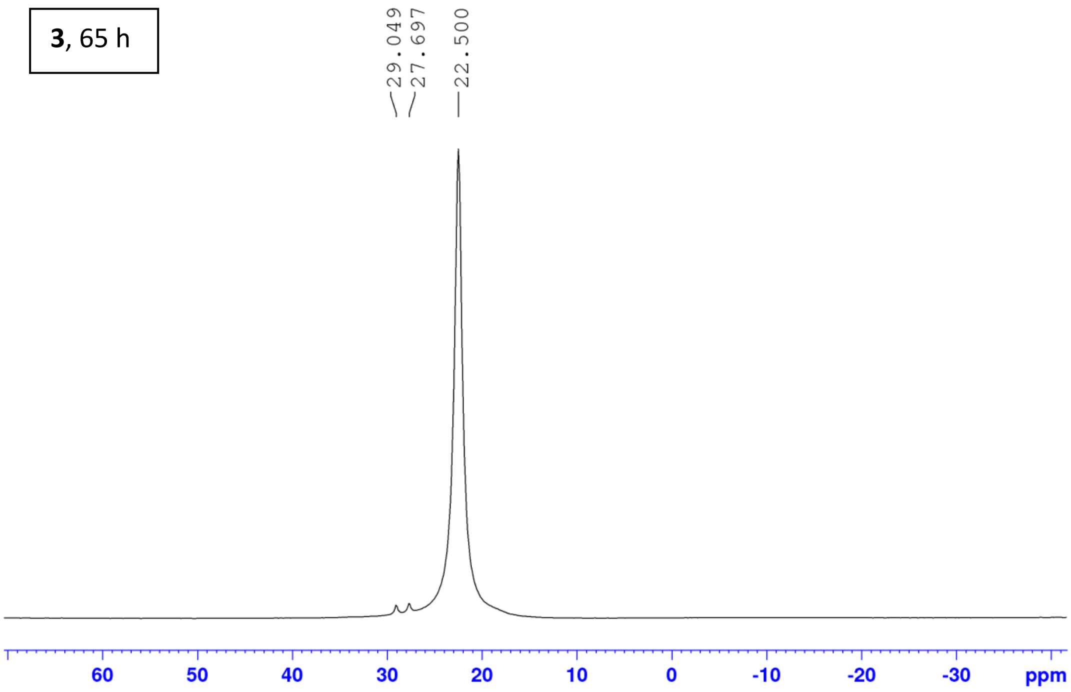

Figure S33: ^{1}H NMR spectra of acetophenone hydroboration using **2, 4, 5** and **6,** in C_6D_6.

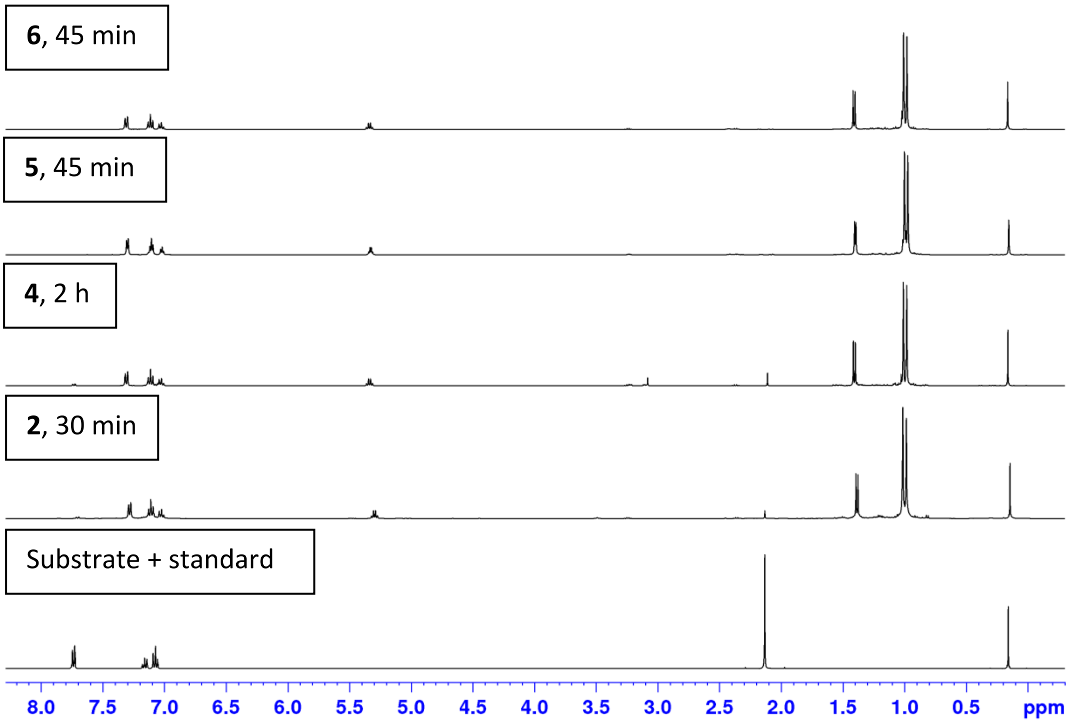

2,2,2-trimethylacetophenone

Figure S34: ^{1}H NMR spectra of 2,2,2-trimethylacetophenone hydroboration using **3** in C_6D_6.

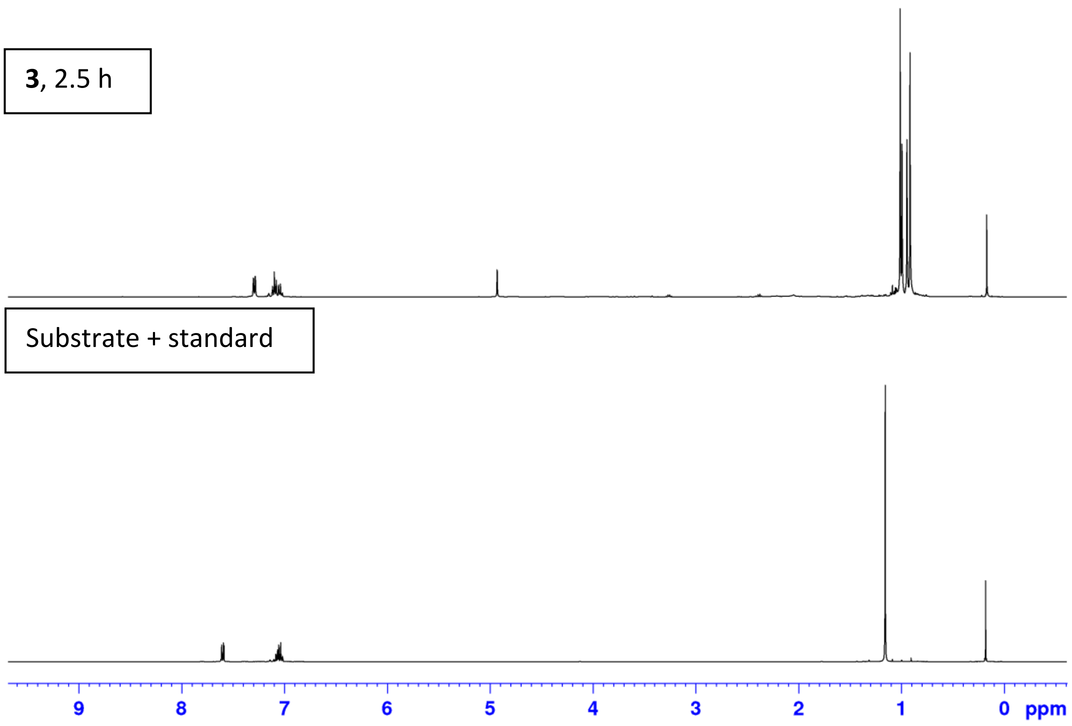

2,2,2-trifluoroacetophenone

Figure S35: ^1H NMR spectra of 2,2,2-trifluoroacetophenone hydroboration using **3** in C_6D_6.

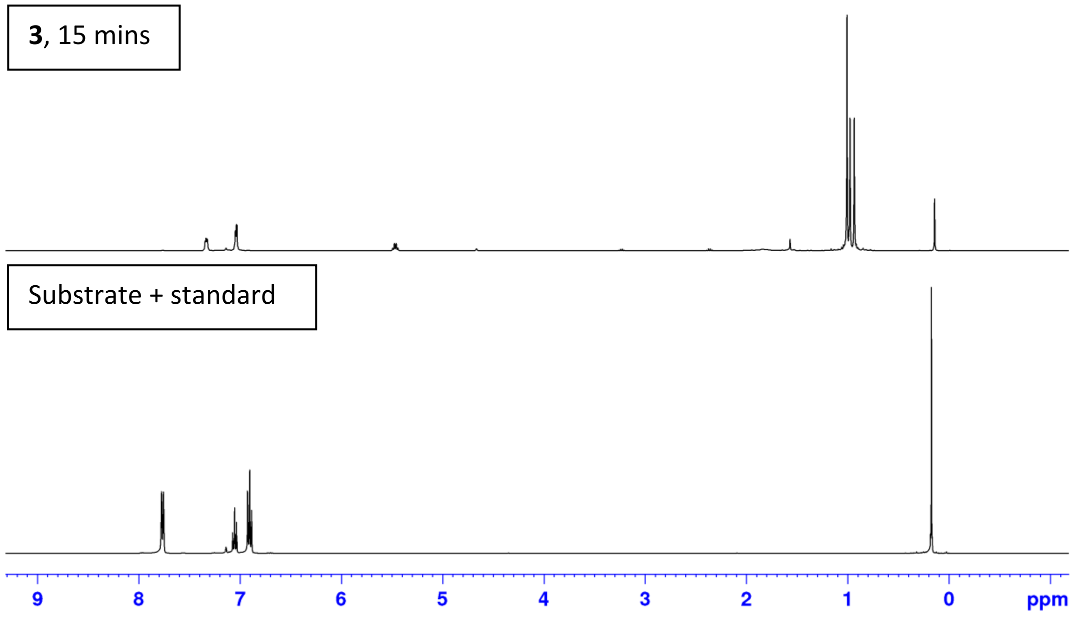

Benzophenone

Figure S36: ^{1}H NMR spectra of benzophenone hydroboration using **1**, **3** or **4** in C_6D_6.

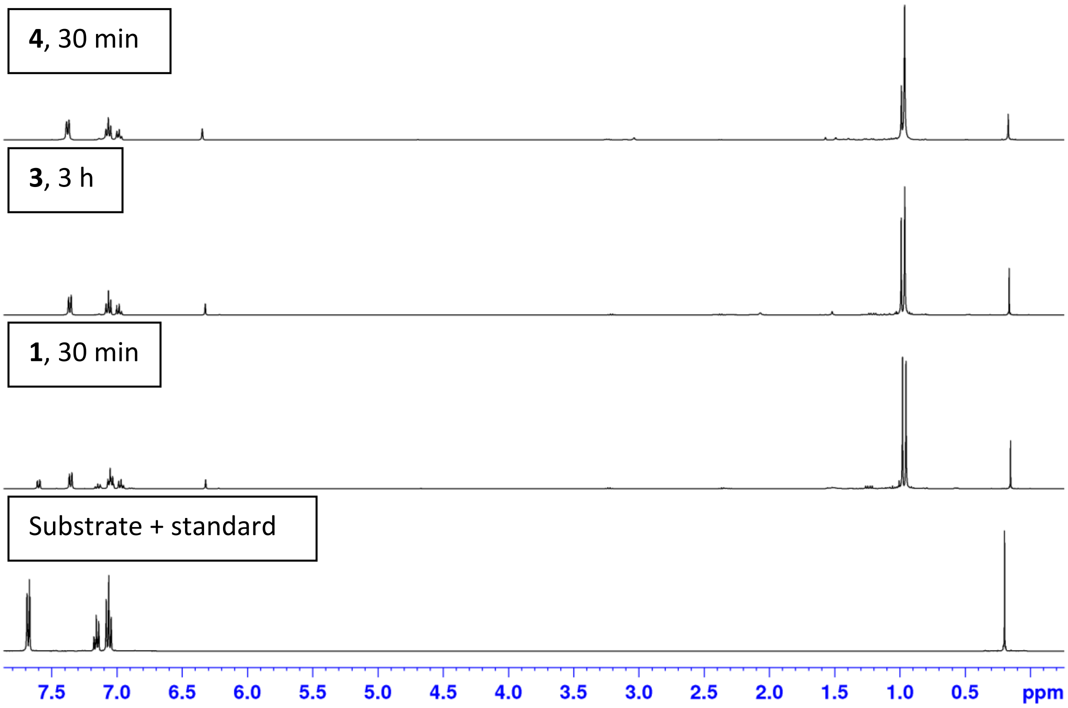

Cyclohexanone

Figure S37: ^{1}H NMR spectra of cyclohexanone hydroboration using **1**, **3** or **4** in C_6D_6.

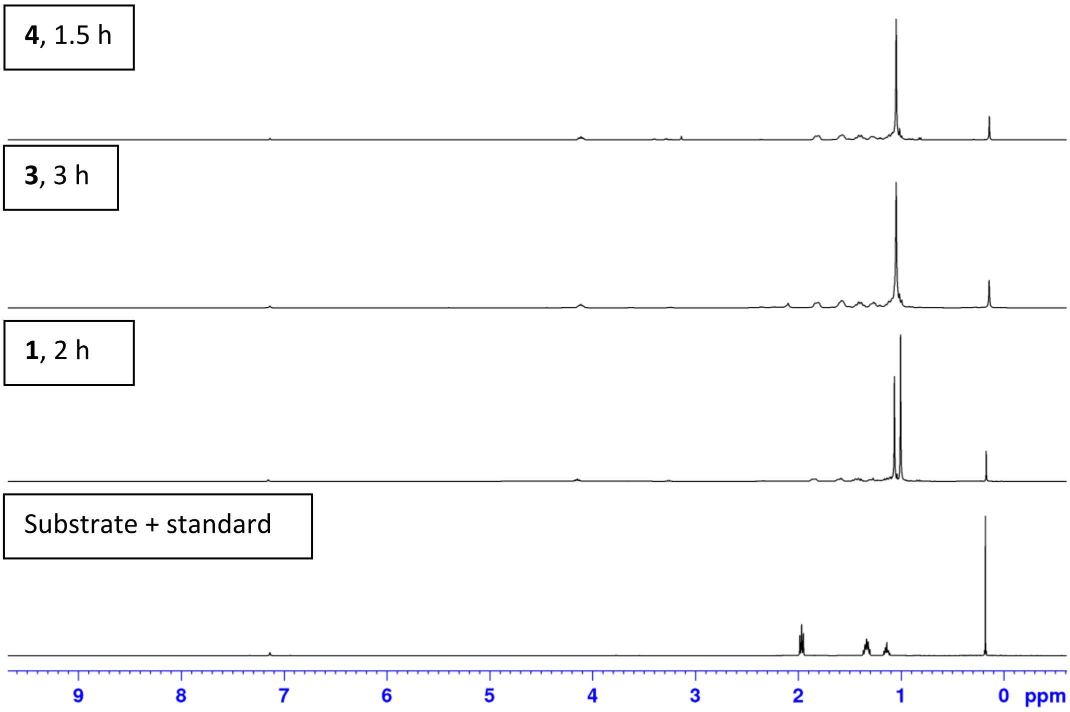

Butan-2-one

Figure S38: ^{1}H NMR spectra of butan-2-one hydroboration using **1**, **3** or **4** in C_6D_6.

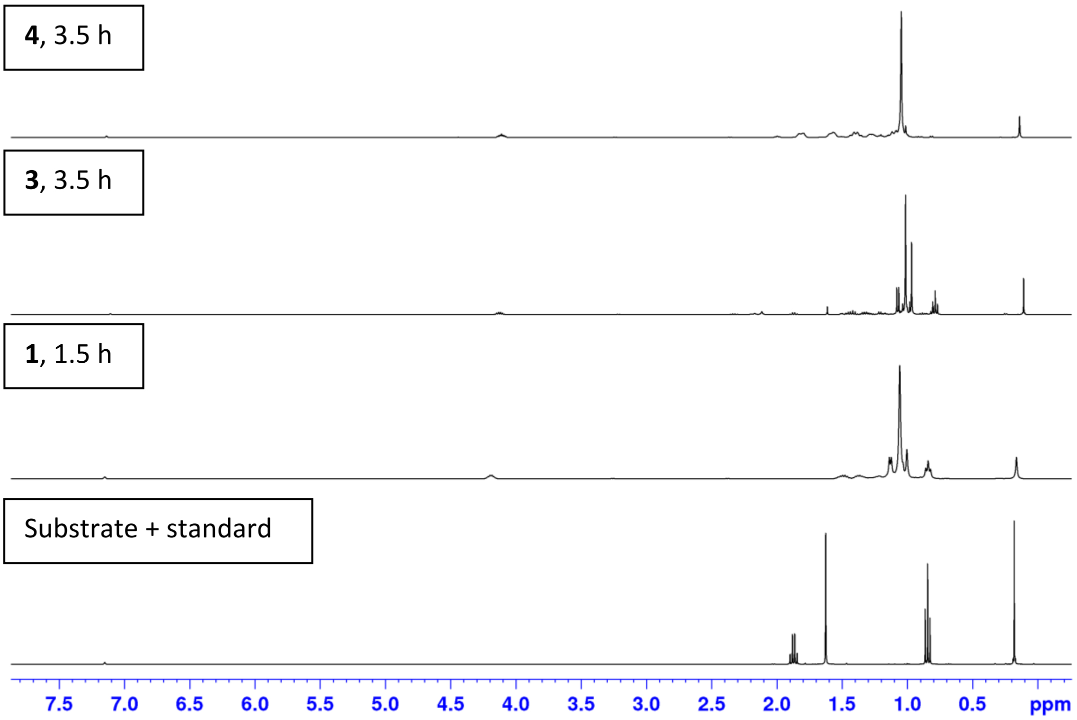

Acetophenone in THF

Figure S39: ^{1}H NMR spectra of acetophenone hydroboration using **1** or **3** or **4** in d_8-THF.

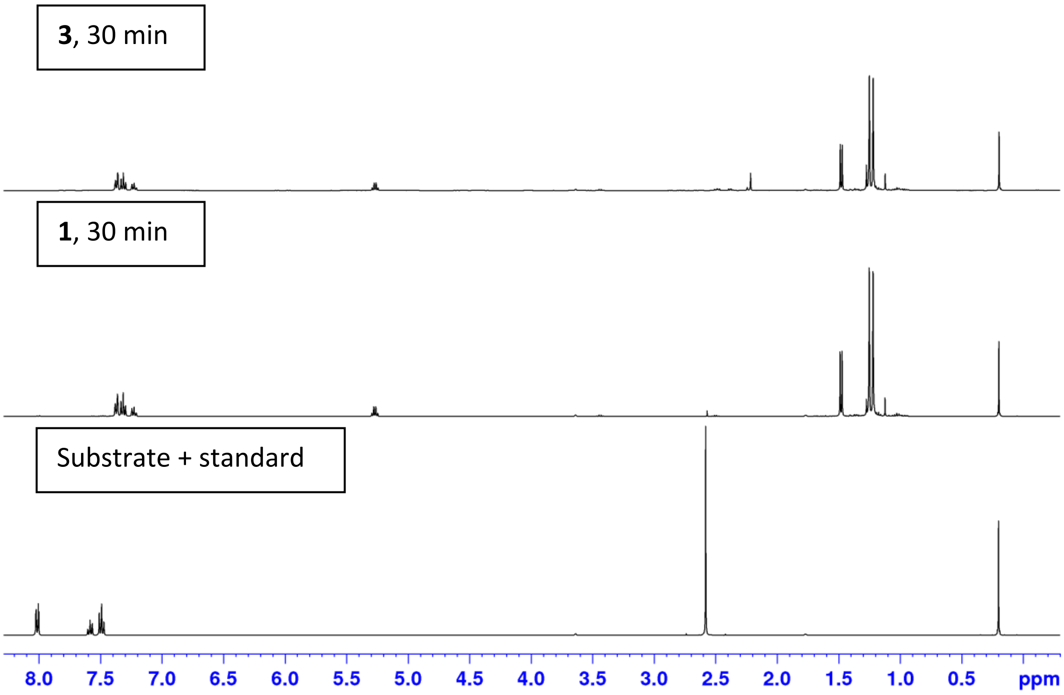

Metallation of 1-Me-1,2,4-triazole using 3 – Synthesis of [PMDETA·LiH(1,2,4-triazolyl)Al(iBu)$_2$] (8)

Solid **3** (185 mg, 0.4 mmol) was suspended in n-hexane (3 mL), resulting in a cloudy white suspension. 1-Me-1,2,4-triazole (0.03 mL, 0.4 mmol) was added and the reaction passed into solution. 1 mL of toluene was added and the reaction was stirred for a further hour at room temperature. Single crystals, suitable for diffraction studies were grown from this solution at -30 °C, in an isolated yield of [PMDETA·LiH(1,2,4-triazolyl)Al(iBu)$_2$] (**8**) (68 mg, 0.17 mmol) 42%.

^{1}H NMR (400.13 Hz, C_6D_6, 298 K) δ 7.75 (s, 1H, triazole), 3.92 (s, 3H, C*H*$_3$), 2.35 (sept, 2H, AlCH$_2$C*H*(CH$_3$)$_2$), 1.95 (s, 3H, C*H*$_3$(PMDETA)), 1.91 (s, 12H, 4 x C*H*$_3$(PMDETA)), 1.72 (br. s, 8H, C*H*$_2$(PMDETA)), 1,39 (2xd, 12H, AlCH$_2$CH(C*H*$_3$)$_2$), 0.61 (dd, 2H, AlC*H*$_2$CH(CH$_3$)$_2$), 0.49 (dd, 2H, AlC*H*$_2$CH(CH$_3$)$_2$) ppm.

^{13}C NMR (100.61 Hz, C_6D_6, 298 K) δ 149.3 (*C*H, triazole), 56.6 (*C*H$_2$(PMDETA)), 53.5 (*C*H$_2$(PMDETA)), 45.4 (*C*H$_3$(PMDETA)), 44.6 (*C*H$_3$(PMDETA)), 37.5 (*C*H$_3$, triazole), 29.4 (AlCH$_2$CH(*C*H$_3$)$_2$), 28.8 (AlCH$_2$CH(*C*H$_3$)$_2$), 28.5 (AlCH$_2$*C*H(CH$_3$)$_2$), 24.5 (Al*C*H$_2$CH(CH$_3$)$_2$)ppm.

^{7}Li NMR (155.47 Hz, C_6D_6, 298 K) δ 0.90 (d, *Li*-H-Al) ppm.

Elemental analysis: *Calculated:* C: 59.38 %, H: 11.46 %, N: 20.77 %. *Found:* C: 58.86 %, H: 11.37 %, N: 20.01 %.

Figure S40: ^{1}H NMR spectrum of **8** in C_6D_6.

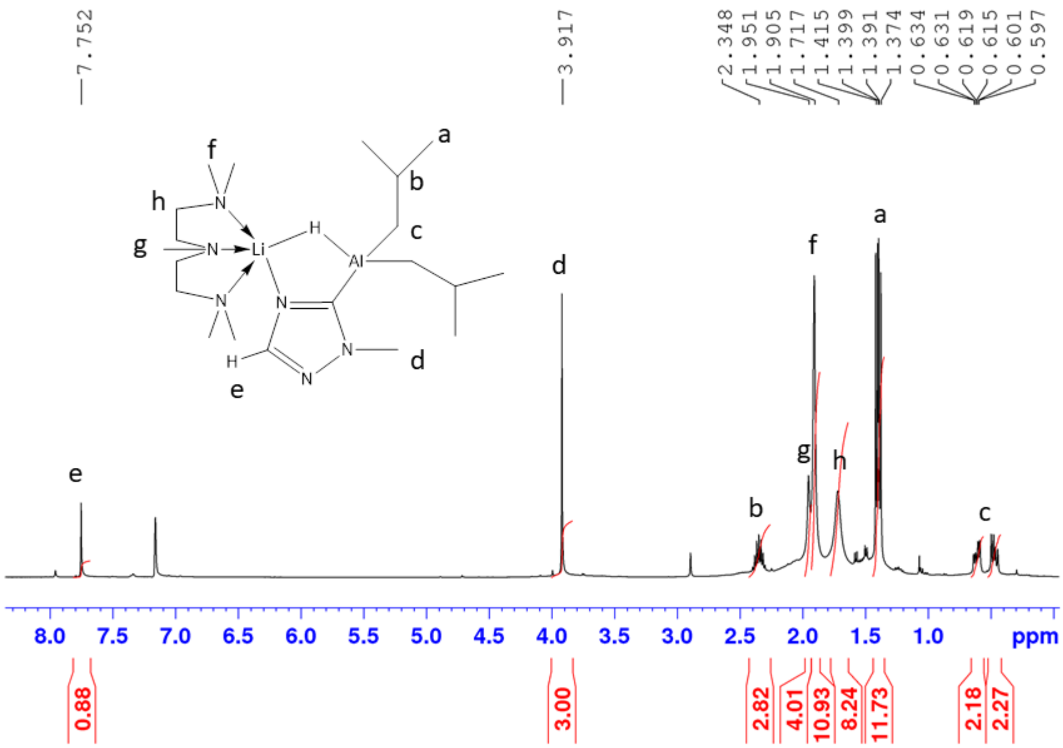

Figure S41: ^{13}C NMR spectrum of **8** in C_6D_6.

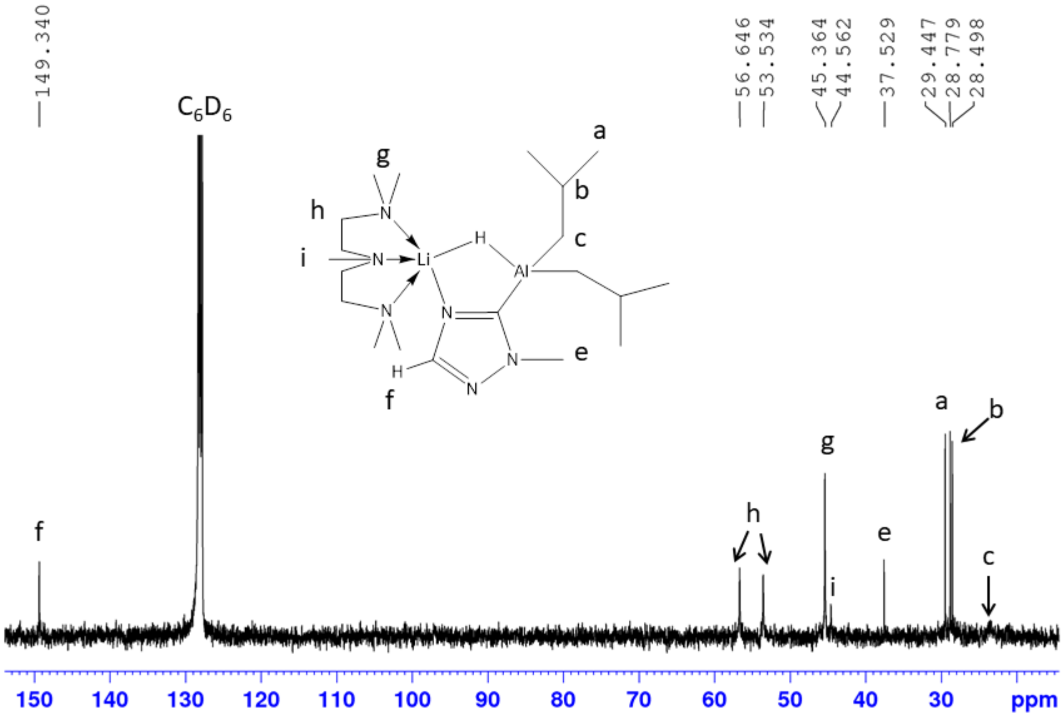

Figure S42: ^{7}Li NMR spectrum of **8** in C_6D_6.

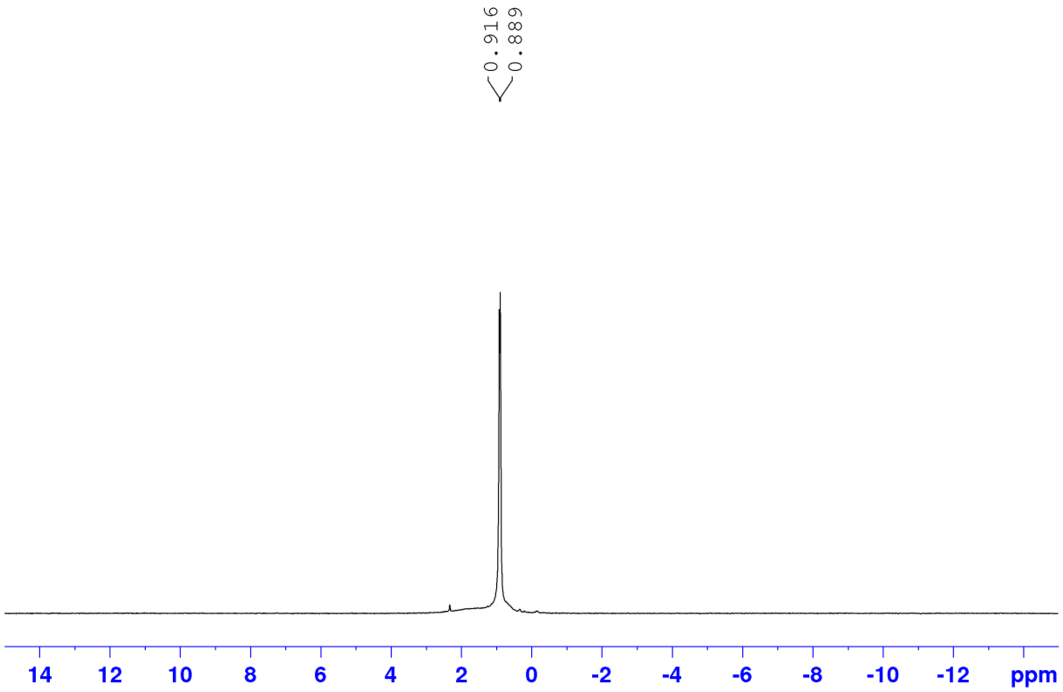

Metallation of 1-Me-1,2,4-triazole using *i*Bu_2AlTMP – Synthesis of [(1,2,4-triazolyl)Al(iBu)$_2$]$_2$ (9)

iBu_2AlTMP (224 mg, 0.8 mmol) was dissolved in toluene (2 mL), then 1-Me-1,2,4-triazole (0.06 mL, 0.8 mmol) was added and the reaction was one hour at room temperature. N-hexane (2 mL) was added and single crystals, suitable for diffraction studies were grown from this solution at -30 °C, affording [(1,2,4-triazolyl)Al(iBu)$_2$]$_2$ (**9**) (55 mg, 0.11 mmol) 31%.

^{1}H NMR (400.13 Hz, C_6D_6, 298 K) δ 7.79 (s, 1H, triazole), 3.53 (s, 3H, CH_3), 1.71 (sept, 2H, $AlCH_2CH(CH_3)_2$), 0.92 (2xd, 12H, $AlCH_2CH(CH_3)_2$), 0.29 (dd, 2H, $AlCH_2CH(CH_3)_2$) ppm.

^{13}C NMR (100.61 Hz, C_6D_6, 298 K) δ 147.0 (*C*H, triazole), 37.1 (*C*H_3, triazole), 27.4 ($AlCH_2CH(CH_3)_2$), 27.3 ($AlCH_2CH(CH_3)_2$), 25.9 ($AlCH_2CH(CH_3)_2$), 21.7 ($AlCH_2CH(CH_3)_2$)ppm.

Elemental analysis: *Calculated:* C: 59.17 %, H: 9.93 %, N: 18.82 %. *Found:* C: 59.11 %, H: 8.94 %, N: 18.34 %.

Figure S43: ^{1}H NMR spectrum of **9** in C_6D_6

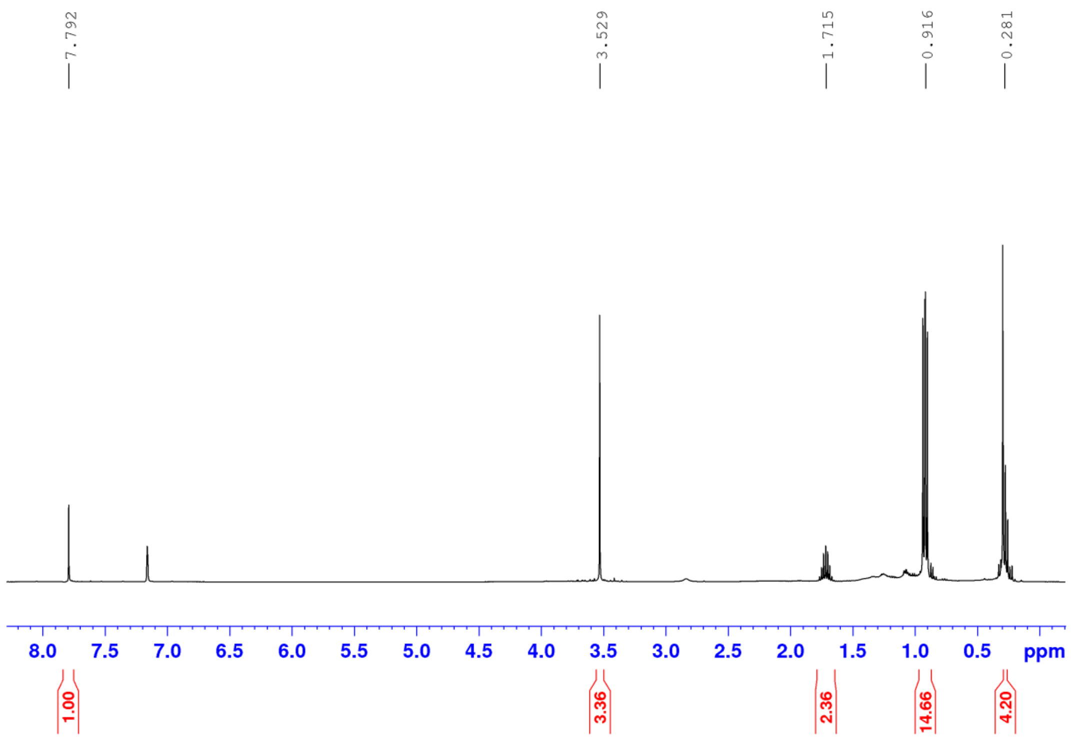

Figure S44: ^{13}C NMR spectrum of **9** in C_6D_6

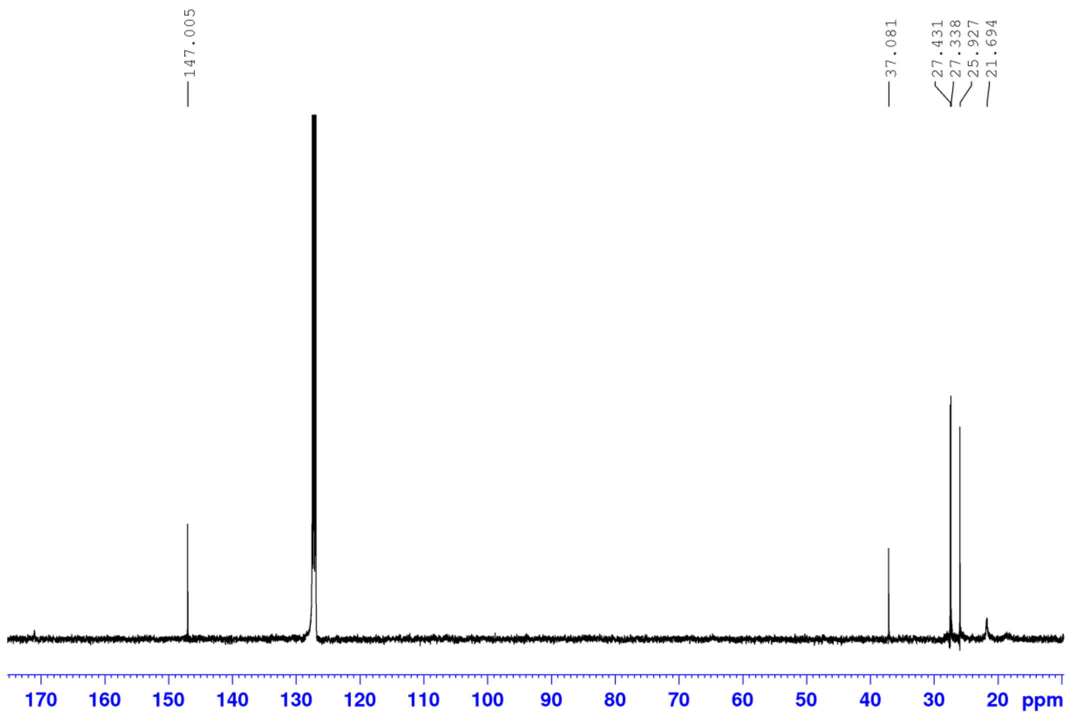

Figure S45: Structure of **9** showing connectivity only.

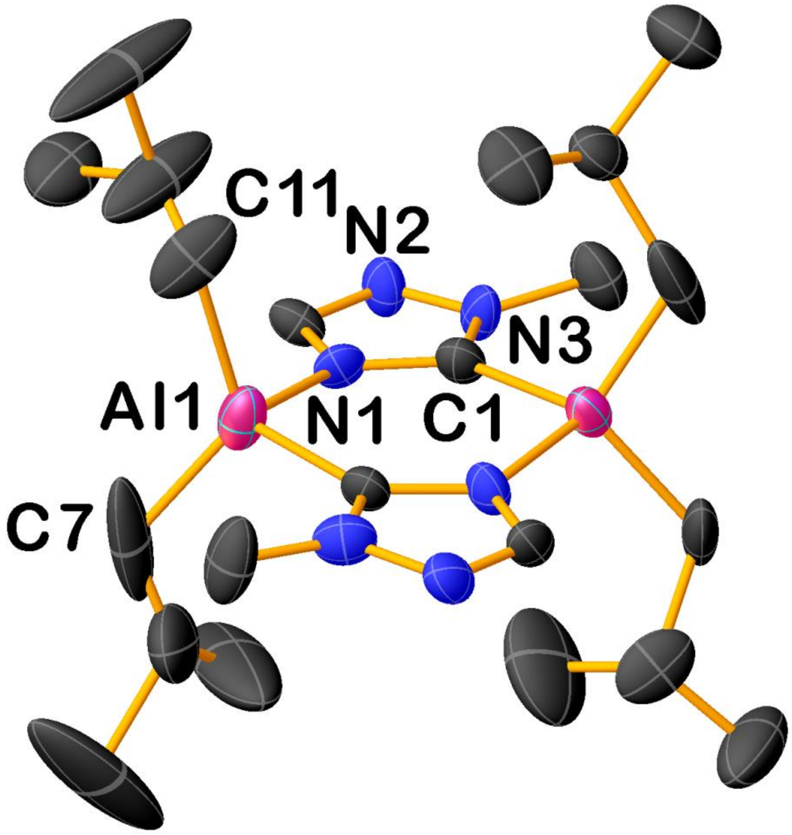

Metallation of Phenylacetylene using 3 – Synthesis of [PMDETA·LiH(C_6H_5CC)Al(iBu)$_2$] (10)

Solid **3** (92 mg, 0.2 mmol) was suspended in n-hexane (4 mL), resulting in a cloudy white suspension. Phenylacetylene (0.02 mL, 0.2 mmol) was added and the reaction passed into solution. 1 mL of toluene was added and the reaction was stirred for a further hour at room temperature. [PMDETA·LiH(C_6H_5CC)Al(iBu)$_2$] (**10**) (48 mg, 0.11 mmol) 57% was isolated by precipitation after concentration of solvent and placing at -30 °C.

^{1}H NMR (400.13 Hz, C_6D_6, 298 K) δ 7.49 (m, 2H, C_8H_5), 7.05 (m, 2H, C_8H_5), 6.97 (d, 1H, C_8H_5), 2.51 (sept, 2H, $AlCH_2C\mathit{H}(CH_3)_2$), 2.05 (s, 3H, $C\mathit{H}_3$(PMDETA)), 1.91 (s, 12H, 4 x $C\mathit{H}_3$(PMDETA)), 1.77 and 1.62 (2xbr. s, 8H, $C\mathit{H}_2$(PMDETA)), 1,52 (2xd, 12H, $AlCH_2CH(C\mathit{H}_3)_2$), 0.63 (m, 4H, $AlC\mathit{H}_2CH(CH_3)_2$) ppm.

^{13}C NMR (100.61 Hz, C_6D_6, 298 K) δ 131.9 (*C*H, C_8H_5), 126.5 (*C*H, C_8H_5), 56.9 ($\mathit{C}H_2$(PMDETA)), 53.2 ($\mathit{C}H_2$(PMDETA)), 45.7 ($\mathit{C}H_3$(PMDETA)), 44.4 ($\mathit{C}H_3$(PMDETA)), 29.2 ($AlCH_2CH(\mathit{C}H_3)_2$), 29.1 ($AlCH_2CH(\mathit{C}H_3)_2$), 28.6 ($AlCH_2\mathit{C}H(CH_3)_2$), 23.8 ($Al\mathit{C}H_2CH(CH_3)_2$)ppm.

^{7}Li NMR (155.47 Hz, C_6D_6, 298 K) δ 0.57 (s, *Li*-H-Al) ppm.

Elemental analysis: *Calculated:* C: 70.72 %, H: 11.39 %, N: 9.90 %. *Found:* C: 69.83 %, H: 11.02 %, N: 9.41 %.

Figure S46: ^{1}H NMR spectrum of **10** in C_6D_6.

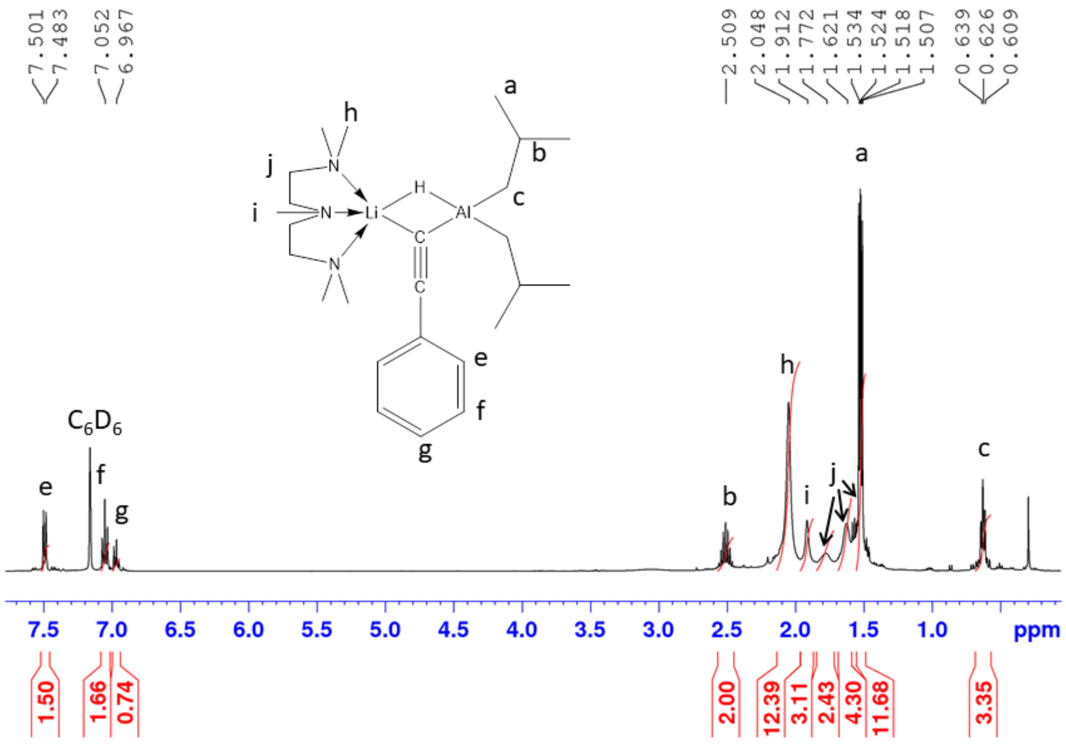

Figure S47: ^{13}C NMR spectrum of **10** in C_6D_6.

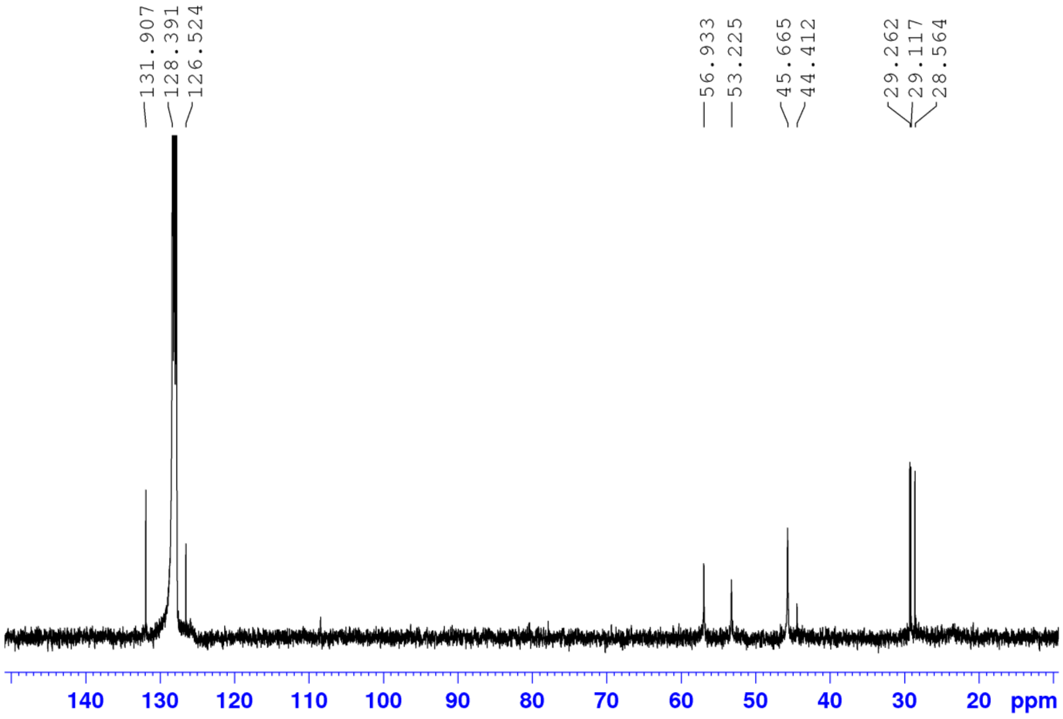

Figure S48: ^{7}Li NMR spectrum of **10** in C_6D_6.

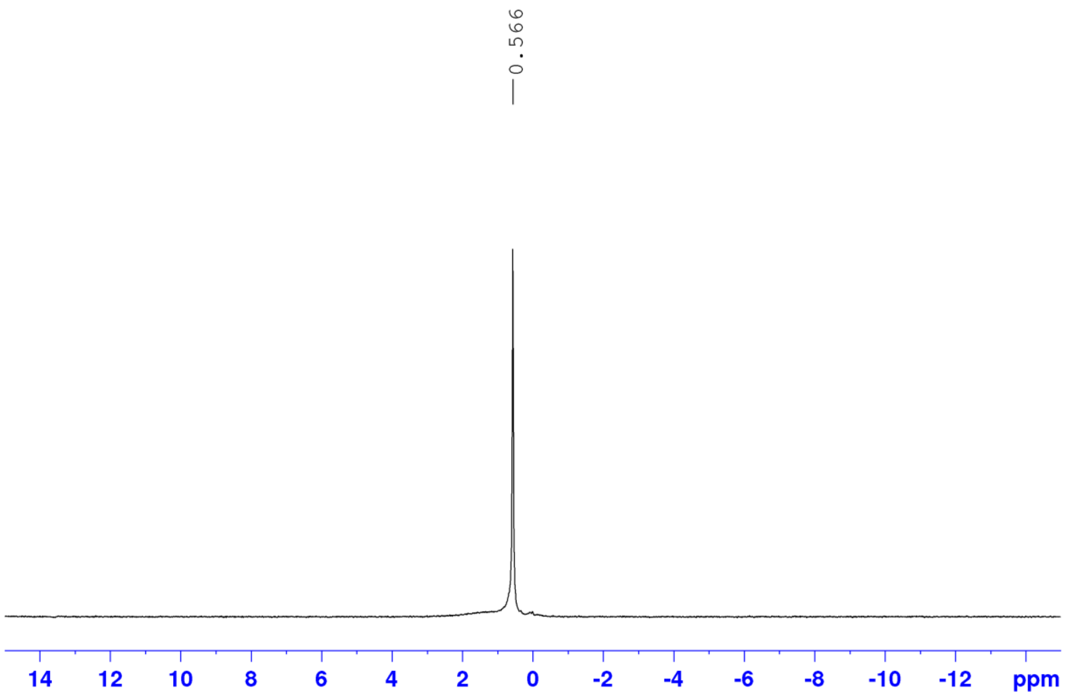

Phenylacetylene

Figure S49: ^{1}H NMR spectra of phenylacetylene hydroboration using **1** in C_6D_6 at 70 °C.

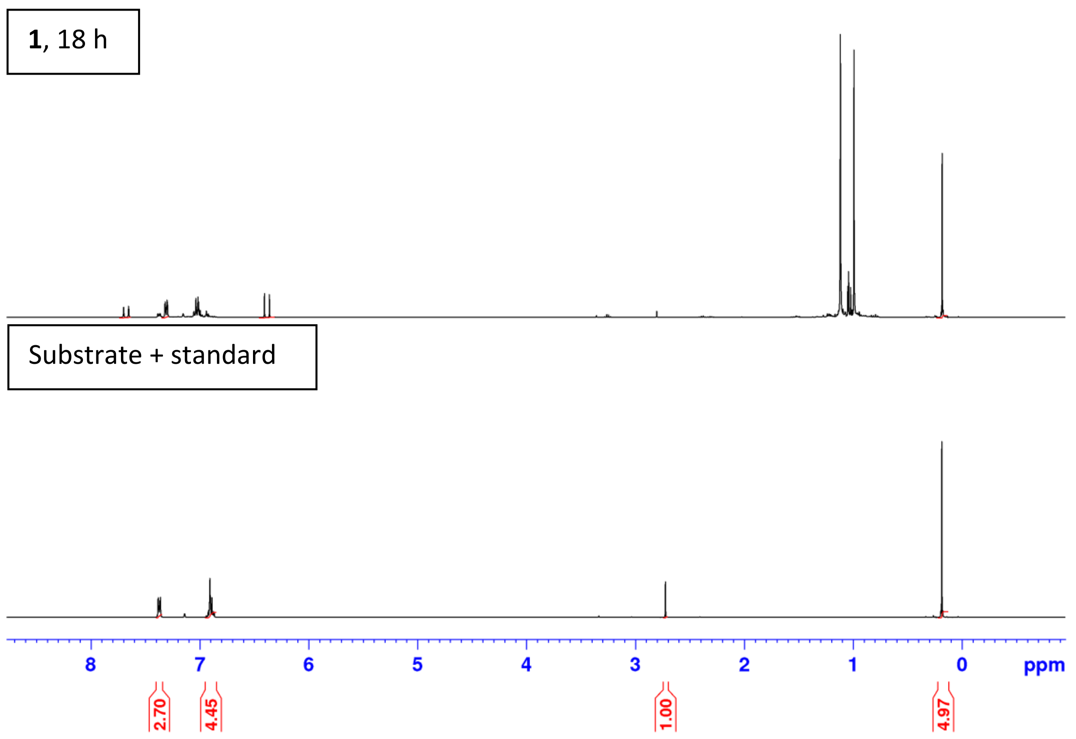

Reaction of pyrazine with 3 – Synthesis of [PMDETA·Li($C_4H_5N_2$)Al(iBu)$_2$TMP] (11)

Solid **3** (139 mg, 0.3 mmol) was dissolved in toluene (4 mL), and solid pyrazine (24 mg 0.3 mmol) was added. The reaction was stirred for 3 hours at room temperature resulting in separation of an orange oil form solution. [PMDETA·Li($C_4H_5N_2$)Al(iBu)$_2$TMP] (**11**) (68 mg, 0.13 mmol) 42% was isolated by precipitation after concentration of solvent and placing at -30 °C. The resulting orange oil is only sparingly soluble in C_6D_6.

^{1}H NMR (400.13 Hz, C_6D_6, 298 K) δ 7.25 (s, 1H, $C_4H_5N_2$), 5.81 (s, 1H, $C_4H_5N_2$), 5.32 (s, 1H, $C_4H_5N_2$), 4.00 (s, 1H, $C_4H_5N_2$), 2.43 (sept, 2H, AlCH$_2$C*H*(CH$_3$)$_2$), 1.99 (br. s, 2H, C*H*$_2$TMP), 1.81 (br. s, 12H, C*H*$_3$TMP), 1.80 (br. s, 4H, C*H*$_2$TMP), 1.56 (br. s, 3H, C*H*$_3$(PMDETA)), 1.54 (s, 12H, 4 x C*H*$_3$(PMDETA)), 1.51 (br. s, 8H, C*H*$_2$(PMDETA)), 1,50 (2xd, 12H, AlCH$_2$CH(C*H*$_3$)$_2$), 0.66 (m, 4H, AlC*H*$_2$CH(CH$_3$)$_2$) ppm.

^{13}C NMR (100.61 Hz, C_6D_6, 298 K) δ 142.1 (*C*H, $C_4H_5N_2$), 119.9 (*C*H, $C_4H_5N_2$), 109.5 (*C*H, $C_4H_5N_2$), 52.9 (*C*H$_2$(PMDETA)), 51.5 (*C*H$_2$(PMDETA)), 45.7 (*C*H$_2$(TMP)), 45.6 (*C*H$_2$, $C_4H_5N_2$), 44.4 (*C*H$_3$(PMDETA)), 43.1 (*C*H$_3$(PMDETA)), 30.1 (*C*H$_3$(TMP)), 29.4 (AlCH$_2$CH(*C*H$_3$)$_2$), 28.7 (AlCH$_2$*C*H(CH$_3$)$_2$), 27.0 (Al*C*H$_2$CH(CH$_3$)$_2$), 18.8 (*C*H$_3$(TMP)) ppm. Assignment facilitated by HSQC experiment

^{7}Li NMR (155.47 Hz, C_6D_6, 298 K) δ 0.47 (s,) ppm.

Figure S49: ^{1}H NMR spectrum of **11** in C_6D_6.

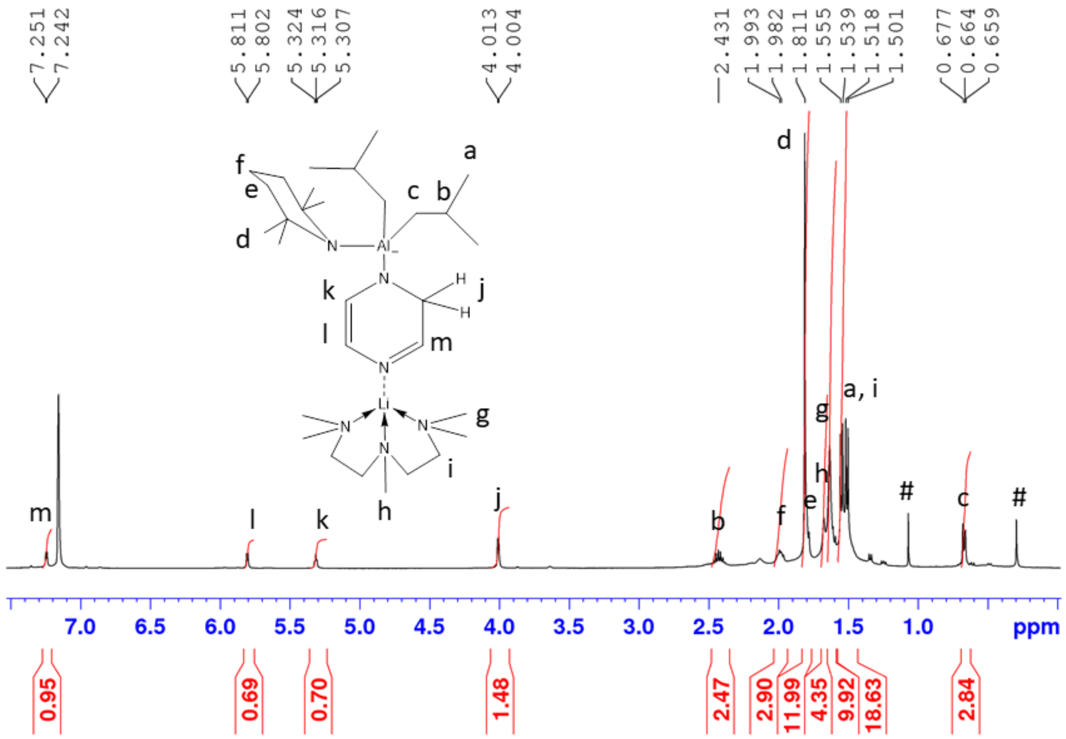

Figure S50: ^{13}C NMR spectrum of **11** in C_6D_6.

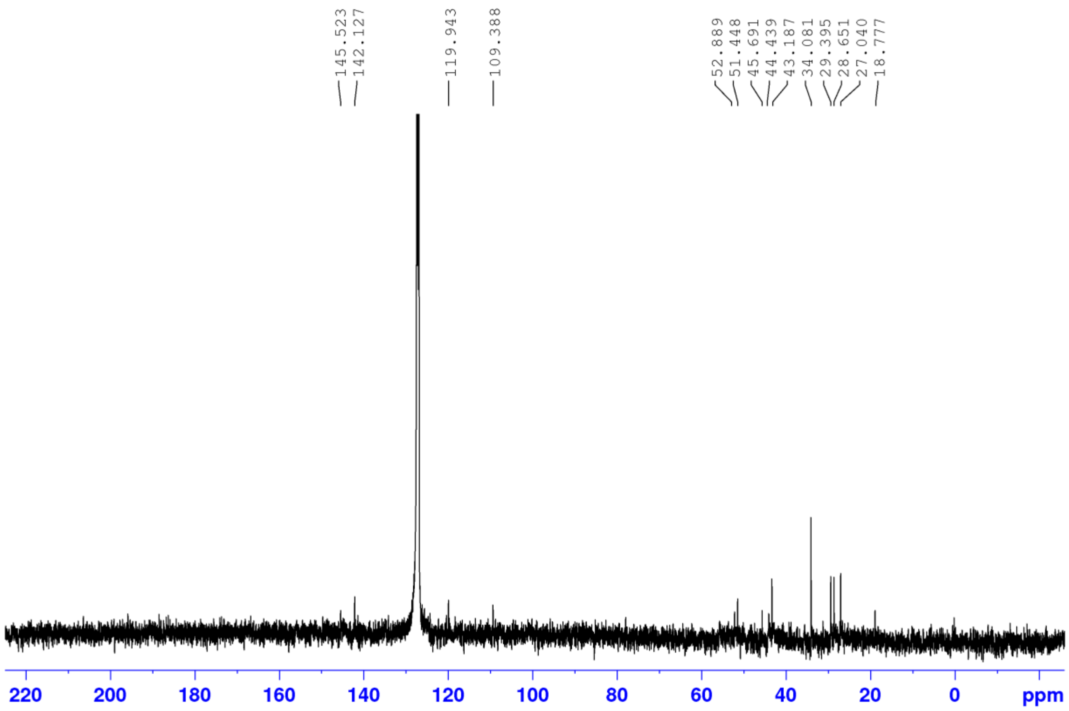

Figure S51: ^{7}Li NMR spectrum of **11** in C_6D_6.

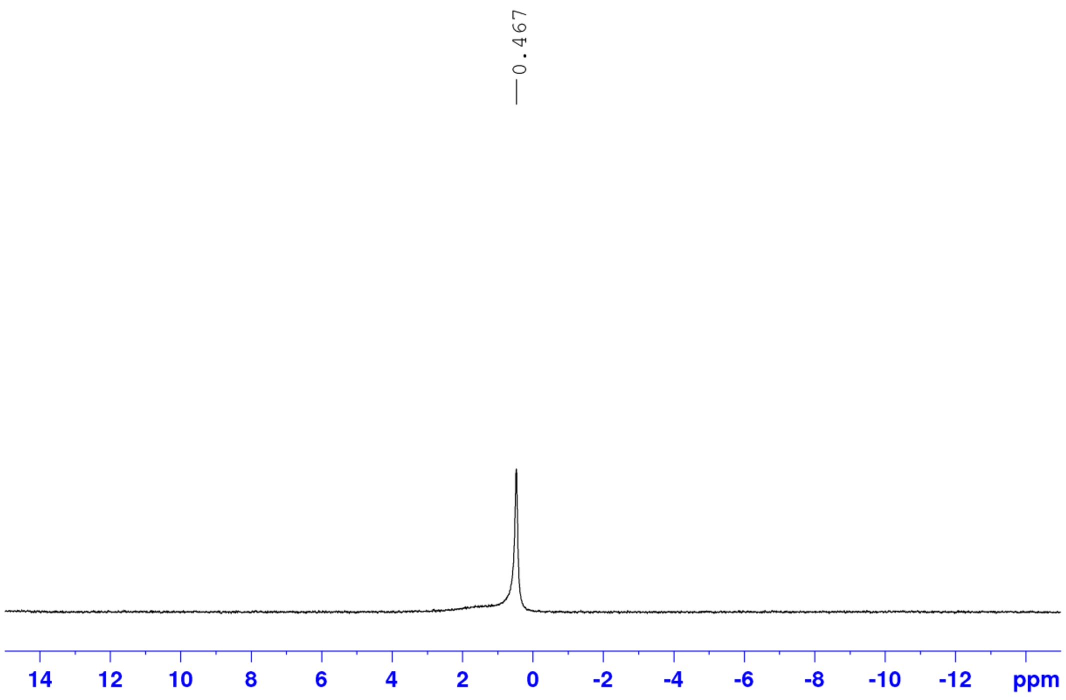

Table S2 **Crystallographic data and refinement details for complexes 2-5.**

	**2**	**3**	**4**	**5**
Empirical formula	$C_{29}H_{61}AlLiNO_3$	$C_{26}H_{60}AlLiN_4$	$C_{46}H_{102}Al_2Li_2N_2O_6$	$C_{43.50}H_{90}Al_2Li_2N_4$
Mol. Mass	505.70	462.7	847.13	737.03
Crystal system	monoclinic	monoclinic	monoclinic	monoclinic
a/ Å	11.1248(5)	19.1684(6)	19.2579(9)	10.2090(5)
b/ Å	16.9065(6)	9.4223(2)	14.5193(6)	15.6410(7)
c/ Å	17.3408(7)	19.1099(13)	19.8917(9)	16.2047(9)
α/ °	90	90	90	90
β/ °	98.668(7)	116.036(3)	104.198(4)	96.543(5)
γ/ °	90	90	90	90
V/ $Å^3$	3244.2(2)	3101.2(3)	5392.0(4)	2570.7(2)
Z	4	4	4	2
λ/ Å	1.54184	1.54184	1.54184	1.54184
Measured reflections	10915	6029	20900	14289
Unique reflections	6017	4366	10182	4859
R_{int}	0.0261	0.0356	0.0656	0.0567
Observed rflns [I>2σ(I)]	4746	4142	7079	3311
GooF	1.085	1.061	1.017	0.958
R [on *F*, obs rflns only]	0.0576	0.0422	0.0795	0.0631
ωR [on F^2, all data]	0.1584	0.1045	0.2677	0.1848
Largest diff. Peak/hole. e/ $Å^{-3}$	0.467 / -0.268	0.325 / -0.192	0.733 / -0.705	0.723 / -0.214

Table S2 (continued) Crystallographic data and refinement details for complexes 6-8.

	**6**	**7**	**8**
Empirical formula	$C_{29.50}H_{59}$ AlLiN$_4$	$C_{42}H_{90}Al_2N_2Na_2O_2$	$C_{20}H_{46}AlLiN_6$
Mol. Mass	503.72	755.10	404.55
Crystal system	triclinic	monoclinic	triclinic
a/ Å	11.8579(12)	15.3850(16)	10.9473(9)
b/ Å	17.1104(16)	17.022(3)	16.559(3)
c/ Å	17.4493(19)	18.6698(19)	16.6206(13)
α/ °	100.517(8)	90	110.674(11)
β/ °	99.835(9)	98.010(9)	90.905(6)
γ/ °	106.697(9)	90	108.419(10)
V/ Å^3	3239.1(6)	4841.6(10)	2646.8(6)
Z	4	4	4
λ/ Å	0.71073	0.71073	0.71073
Measured reflections	27653	33527	23439
Unique reflections	13840	11237	11501
R_{int}	0.0288	0.0648	0.0325
Observed rflns [I>2σ(I)]	9166	7073	7840
GooF	1.014	1.023	1.017
R [on *F*, obs rflns only]	0.0697	0.0598	0.0601
ωR [on F^2, all data]	0.2012	0.1742	0.1704
Largest diff. Peak/hole. e/ Å^{-3}	0.655 / -0.372	0.401 / -0.293	0.431 / -0.214

References

1) (a) G. M. Sheldrick, *Acta Crystallogr.* **2008**, *A64*, 112–122; (b) G. M. Sheldrick, *Acta Crystallogr.* **2015**, *C71*, 3–8.

(2) O. V. Dolomanov, L. J. Bourhis, R. J. Gildea, J. A. K. Howard, H. Puschmann, *J. Appl. Crystallogr.* **2009**, *42*, 339–341.
